# Behavior Change Interventions to Promote Healthy Lifestyles Among Adolescent and Young Adult Childhood Cancer Survivors: A Scoping Review

**DOI:** 10.1002/cnr2.70721

**Published:** 2026-10-18

**Authors:** C. Gélinas‐Gagné, K. Bilodeau, N. Guarascio, M. Caru, H. Coltin, D. Curnier, L. Desjardins, C. Laverdière, V. Marcil, R. Santiago, S. Vairy, S. Sultan

**Affiliations:** ^1^ Department of Psychology Université de Montréal Montréal Quebec Canada; ^2^ Azrieli Research Center CHU Sainte‐Justine Montréal Quebec Canada; ^3^ Faculty of Nursing Science Université de Montréal Montréal Quebec Canada; ^4^ Hôpital Maisonneuve‐Rosemont Research Center Montréal Quebec Canada; ^5^ Department of Pediatrics, Division of Hematology and Oncology Penn State College of Medicine Hershey Pennsylvania USA; ^6^ Department of Public Health Sciences Penn State College of Medicine Hershey Pennsylvania USA; ^7^ Department of Pediatrics Université de Montréal Montréal Quebec Canada; ^8^ School of Kinesiology and Physical Activity Sciences, Faculty of Medicine Université de Montréal Montréal Quebec Canada; ^9^ Sainte‐Justine University Health Center Montréal Quebec Canada; ^10^ Department of Nutrition Université de Montréal Montréal Quebec Canada; ^11^ CHUS Fleurimont Sherbrooke Quebec Canada; ^12^ Department of Pediatrics, Faculty of Medicine Université de Sherbrooke Sherbrooke Quebec Canada

**Keywords:** adolescent and young adult childhood cancer survivors, alcohol use, behavior change, childhood cancer, lifestyle intervention, nutrition, physical activity, scoping review, tobacco use

## Abstract

**Background:**

Adolescent and young adult childhood cancer survivors (AYA‐CCS) face elevated risks of chronic health conditions and premature mortality further exacerbated by poor lifestyle behaviors including physical inactivity, poor nutrition, and substance use. Behavioral interventions are essential for supporting long‐term health in this population; however, the evidence remains fragmented and unbalanced across lifestyle domains. This scoping review aimed to identify and describe behavioral interventions promoting healthy lifestyles among AYA‐CCS, focusing on four domains (physical activity, nutrition, alcohol use, and tobacco use), and to map the behavior change techniques employed. Following the Joanna Briggs Institute methodology, a comprehensive search was conducted. Eligible studies targeted AYA‐CCS aged 13–39 years and included interventions addressing at least one of the four behavioral domains. Records were published between 2005 and 2025. Data were extracted using the Template for Intervention Description and Replication (TIDieR) checklist and categorized according to the Behavior Change Wheel to identify intervention functions and sources of behavior change.

**Findings:**

The search identified 6170 records, 23 articles met inclusion criteria (representing 17 distinct interventions). Most interventions (76.5%) targeted physical activity. Education, enablement, and training were the most frequently used intervention functions, primarily addressing psychological capability and reflective motivation. Strategies targeting automatic motivation, physical capability, or social opportunity were far less common. Only three multi‐domain interventions were identified.

**Conclusion:**

Current behavior change interventions for AYA‐CCS are dominated by physical activity programs, with limited attention to nutrition, alcohol, and tobacco use. While many interventions were theory‐informed, important gaps remain in fidelity reporting, multi‐behavioral targeting, and explaining mechanisms of change.

## 1 Introduction

Adolescent and young adult childhood cancer survivors (AYA‐CCS) face a distinctive set of challenges. While AYA‐CCS are expected to live for many decades beyond their diagnosis [1], they often experience chronic health conditions [2] and face a higher risk of premature death compared to their peers [2, 3]. Childhood cancer survivors (CCS) experience a wide range of late and long‐term effects, impacting multiple aspects of health [3], with repercussions on nearly every organ and system [4]. Despite this important vulnerability, they engage in risk‐taking behaviors at rates comparable to non‐clinical peers [1]. Supporting AYA‐CCS in acquiring and maintaining healthy lifestyles is essential, with behavioral interventions playing a pivotal role in this effort [5, 6, 7].

Few health behavior programs have been developed and refined for CCS [8], despite the fact that modifiable risk factors, such as physical inactivity, poor nutrition, and alcohol and tobacco use, can significantly exacerbate long‐term treatment‐related toxicities [9]. Promoting healthy lifestyles early, particularly during a crucial period for physical development and the formation of lifelong habits [10, 11], may improve long‐term health outcomes for cancer survivors [12]. As adolescents and young adults gain greater autonomy, they should therefore become a key target group for behavioral interventions.

Studies frequently combine AYA cancer survivors with pediatric or adult cancer populations, often overlooking the unique characteristics of each group [13]. In the present study we chose to define AYA‐CCS as individuals aged 13 to 39 [14] who were diagnosed with childhood cancer, that is, cancer diagnosed before the age of 21 or a typical form of childhood cancer (e.g., leukemia, lymphoma, neuroblastoma, sarcoma) [15] with onset before age 30 years [16].

To our knowledge, this is the first review to synthesize health promotion interventions in AYA‐CCS. Previous reviews have synthesized findings in cancer survivors, Pugh et al. [17], Vasilopoulou et al. [18] and Coffman et al. [19] included survivors diagnosed during adolescence or young adulthood but excluded those diagnosed in childhood. On the other hand, Stolley et al. [20] included interventions for CCS without specifying age at the time of the intervention. Other reviews have focused on more specific populations or stages of the cancer trajectory. For example, Morales et al. [21, 22] synthesized evidence on physical activity interventions for pediatric patients undergoing cancer treatment. Among childhood cancer survivors, Ha et al. [23] reviewed physical activity interventions specifically in children survivors, whereas Mizrahi et al. [24] examined physical activity interventions across both childhood and adult survivors of childhood cancer. Together, these reviews highlighted that most existing interventions have focused primarily on physical activity, with insufficient attention to other critical aspects of health such as nutrition and substance use [17, 18, 20]. They also underscore the need for greater research attention to interventions specifically designed for AYA‐CCS, whose developmental and survivorship needs may differ from those of younger children or older adults. Coffman et al. [19] examined the behavior change techniques used in populations diagnosed during adolescence and young adulthood; however, no studies have yet explored interventions through a behavior change lens in CCS.

In public health, four behavioral domains are identified as the four most important modifiable behavioral risk factors for non‐communicable diseases and all‐cause mortality [25]: physical activity, nutrition, alcohol use, and tobacco use. Physical activity is widely recognized for positively influencing an array of health outcomes, including cardiovascular health, insulin sensitivity, muscle mass, weight management, and lowering morbidity and mortality [26]. Among CCS, regular activity improves cardiovascular outcomes and survival [27], yet they are typically less active [28], less fit [29], and more often overweight or obese than their peers [30, 31]. Similarly, a balanced diet is widely recognized to help prevent cardiovascular disease [32], Type 2 diabetes [33], obesity [34] and all‐cause mortality [35]. CCS often show poor dietary habits, including low whole grain and nutrient intake, high sodium consumption, and excessive empty calories [36], with about half of survivors' daily energy frequently coming from ultra‐processed foods [37]. Alcohol consumption increases risks for all‐cause mortality [38] and morbidity, including cardiovascular diseases [39], liver cirrhosis [40], and cancers [41]. CCS are more likely than their peers to engage in risky drinking [42]. Smoking is a significant risk factor for early and all‐cause mortality [43, 44] and increases the likelihood of developing cardiovascular diseases [45] and respiratory diseases [46]. It is believed to be the cause of about 20% of all cancers [47]. Tobacco use among CCS heightens the risk of poor health outcomes [9], such as inflammation, oxidative stress, and vascular damage, placing survivors at particularly high risk of severe complications [48]. In CCS, the prevalence of smoking remains between 10% and 22%, similar to that of their peers [42, 49].

In sum, establishing lifelong physical activity habits early is essential to reducing treatment‐related late effects and the risk of obesity [50]. Proper nutrition plays a key role in preventing secondary cancers and supporting long‐term health outcomes [51]. Encouraging moderation or abstention from alcohol is also critical for reducing health risks and improving long‐term well‐being among CCS [52, 53]. Finally, fostering smoke‐free habits early in life is vital, as smoking during adolescence strongly predicts smoking behavior in adulthood and poses significant risks for this already vulnerable population [43].

AYA‐CCS have been largely overlooked in health behavior research [17], leaving critical gaps in our understanding of effective lifestyle interventions for this group. Few studies have identified interventions specifically tailored to their age and diagnostic context, despite their distinct developmental and clinical needs. To date, no review has systematically examined the four key health behavior domains in AYA‐CCS. There is also limited knowledge of how such programs are defined and evaluated, and even less on specific techniques used to support and sustain behavior change. Addressing these gaps is essential to inform the development of targeted, evidence‐based interventions that can improve long‐term health outcomes in this vulnerable population. This scoping review aims to identify behavioral interventions designed to promote healthier lifestyles among AYA‐CCS, with a focus on four domains: physical activity, nutrition, alcohol use and tobacco use. Specifically, we aim to: (1) list and describe all interventions across the four domains, examining their characteristics, activities and formats; and (2) identify which behavior change techniques these interventions use to promote healthier lifestyles across the four behavior domains and how authors expect these techniques to generate change, that is, their explicit or implicit model of change. By identifying and describing available interventions across these domains, this review will provide an overview of current approaches and highlight gaps in intervention development and reporting. Furthermore, examining the behavior change techniques and underlying models of change used in these interventions will help clarify the mechanisms through which interventions are expected to promote healthier lifestyles and inform the development of future evidence‐based interventions tailored to AYA‐CCS.

## 2 Methods

This scoping review was conducted in accordance with the Joanna Briggs Institute (JBI) methodology [54]. A research protocol was deposited on February 25, 2025 (DOI: https://doi.org/10.5683/SP3/JE28ZJ). Along the research process, we made slight changes to the protocol in relation to the age range of participants and the type of items included (File S1).

### 2.1 Inclusion/Exclusion Criteria

#### 2.1.1 Inclusion Criteria

##### 2.1.1.1 Participants

The scoping review included articles interested in AYA‐CCS, defined as individuals aged 13 to 39 who were diagnosed with a childhood cancer (diagnosed before the age of 21 or a typical form of childhood cancer [e.g., leukemia, lymphoma, neuroblastoma, sarcoma]) [15] with an onset earlier than age 30 years. All types of cancer were considered. This definition reflects the overlap in disease types across pediatric and young adult oncology and aligns with AYA frameworks that define this population through cancer type and developmental context alongside age at diagnosis [55, 56, 57, 58, 59]. Ultimately, this approach captures survivors of cancers with pediatric‐type biology and shared treatment pathways across the AYA spectrum.

We included interventions that met one or more of the following criteria; the intervention was explicitly designed for adolescents and/or young adults (13–39 years), whether in its objectives, content, or theoretical rationale; the text clearly stated that the intervention targeted young adults, or used terms such as *emerging adults* or *young adults* in a context consistent with the 13–39 age range; and the study sample was primarily composed of participants aged 13–39 years. In cases where studies included broader or mixed age ranges, inclusion was based on whether AYA‐CCS were explicitly represented and whether intervention characteristics could be extracted as relevant to this subgroup.

##### 2.1.1.2 Concept

The review considered studies reporting on interventions designed to induce behavior change in AYA‐CCS. Interventions targeting lifestyles or behaviors related to physical activity, nutrition, alcohol use, and tobacco use will be included. This review included articles that addressed health behavior as either a primary, secondary, or tertiary outcome.

##### 2.1.1.3 Context

This review was not limited to specific subcultural factors, geographic locations, timeframe, racial or gender‐based interests, or to a specific setting.

##### 2.1.1.4 Types of Sources

This review included both quantitative and qualitative designs, and articles written in English or French. Research protocols were also included as they often provide detailed descriptions of intervention components, delivery formats, and theoretical frameworks that are not always fully reported in completed outcome publications.

No publication date limits were set.

#### 2.1.2 Exclusion Criteria

##### 2.1.2.1 Participants

We excluded studies that met any of the following criteria: the sample was primarily composed of participants outside the 13–39 age range; the intervention was designed for a general adult population without adaptation or specific intent toward AYAs; the report provided did not indicate that the needs or characteristics specific to AYAs were considered in the design or implementation of the intervention.

##### 2.1.2.2 Concepts

We excluded articles that focused exclusively on the physiological outcomes of lifestyle interventions without addressing the behavioral components of health habits. For example, studies reporting solely on the cardiometabolic effects of interventions were not included.

##### 2.1.2.3 Types of Sources

Clinical trials, conference abstracts, case reports, short communications, theses, and letters to the editor were excluded, as they typically lack sufficient detail on interventions to support data extraction that adequately addresses the research question.

### 2.2 Search Strategy

Two independent reviewers (CGG and NG) conducted a search of the literature to assess the scientific evidence for each study. Our search strategy included manually scanning the references of all identified articles for additional studies. The keywords and MeSH terms used (File S2) were validated and adapted by a professional academic librarian (see acknowledgments). The search strategy was adapted for each database. An initial set of keywords was developed and used across all databases. For databases that support controlled vocabulary, MeSH terms were incorporated alongside keywords (in title and abstract) to improve retrieval sensitivity. In databases without controlled vocabulary indexing, searches were conducted using keywords only, with syntax and terminology adjusted to suit each database. Literature searches were performed in the following electronic databases: MEDLINE (PubMed), PsycINFO (APA, PsychNET), CINAHL (EBSCO), Embase (Elsevier), and Cochrane Database of Systematic Reviews (Cochrane Library).

### 2.3 Study Selection

The initial search was completed on February 5, 2025 and was rerun on July 6, 2026 using the same strategy and eligibility criteria. Records identified from the search were compiled and imported into Covidence [60]. Two independent members of the research team (CGG and NG) reviewed titles, abstracts, and keywords to identify and exclude any additional duplicates and verify eligibility. In the event of disagreements between the two reviewers, a third reviewer (SS) was consulted. Items with titles and abstracts deemed relevant by the review team were retrieved in full. The same team assessed the full‐text documents for eligibility via Covidence, following the predefined inclusion criteria. When a full‐text citation did not meet these criteria, a reason for exclusion was documented. The search results are presented in line with the Preferred Reporting Items for Systematic Review and Meta‐Analyses Extension for Scoping Reviews (PRISMA‐ScR) guidelines and will be illustrated using a PRISMA‐ScR flow diagram and checklist [61] (File S3). The search update yielded one additional eligible record [62]. This report contributed to the descriptive synthesis and did not alter the overall interpretation of the findings. Consistent with PRISMA‐S guidance [63], records identified from the original and updated searches were aggregated and reported as combined totals in the PRISMA flow diagram.

### 2.4 Data Extraction

The data collection process for eligible full‐text articles was carried out by two independent reviewers (CGG. and NG), with validation by a third author (SS). We extracted the following characteristics: authors and publication year, participant details (including number of participants, age at diagnosis, age at the time of the study, gender or sex, and types of cancer), study design, aims of the study, targeted behaviors, primary, secondary, or tertiary outcomes, measures used, main findings, and conclusions. Categorization of primary and secondary outcomes followed the authors' classifications if available. When not available, outcomes status was interpreted based on the study's stated objectives. Conclusions were drawn according to the studies' objectives. Intervention functions and COM‐B sources, based on the Behavior Change Wheel (BCW) [64] were identified using a structured coding approach. Detailed descriptions of intervention content were extracted from each study and mapped to the COM‐B source of behavior and intervention functions using established definitions and exemplar materials described by Michie et al. [64] to promote standardized interpretation of constructs. An intervention function or COM‐B source was coded only when the reported intervention contained an explicit component or action consistent with the corresponding definition or example provided by Michie et al. [64]; when the intervention description was insufficient to support a classification, the category was not assigned. All included studies were independently coded by two reviewers (CGG. and NG). Our coding matrix included 17 interventions, with each intervention coded across 15 elements (9 intervention functions and 6 COM‐B sources), resulting in 255 coding entries. Discrepancies occurred in about 13% of entries and were resolved through discussion. When consensus could not be reached, we used arbitration by a third reviewer (SS) in 1.6% of total entries. Following reconciliation, the complete dataset underwent a final validation review to ensure consistency and coherence of the coding across all included studies, thereby enhancing the transparency and reproducibility of the mapping process.

Research protocols often provide detailed descriptions of intervention components, delivery formats, and theoretical frameworks that are not always fully reported in completed outcome publications. In the present study, they were used exclusively for descriptive purposes. They did not provide outcome data and were therefore not included in any synthesis or interpretation of intervention impact. Where a protocol corresponded to a completed study, it was not counted separately in relation to evidence volume or outcomes; instead, it was used to complement the description of the intervention design and theoretical content.

The research team completed the data extraction process manually. As a final quality assurance step, the completed data extraction table was cross‐checked for internal consistency using NotebookLM [65]. The tool was used exclusively as a post‐data extraction quality assurance aid to identify potential inconsistencies within the completed extracted data. The tool was not used to extract data from source documents, interpret study findings, or generate conclusions. The few suggestions produced by the tool were independently verified against the original full‐text articles by the review team, and no AI‐generated content was directly incorporated without human confirmation. As a result of this process, only one record [66] was slightly modified following re‐review of the original publication, as noted in Table 2.

### 2.5 Data Presentation

For our first objective, we extracted information about the intervention presented in each study. We used the Template for Intervention Description and Replication (TIDieR) checklist, a well‐established tool for ensuring transparency in intervention reporting [67]. Although originally designed to guide authors in describing research interventions, it has also been used as an extraction tool in systematic reviews [68]. The TIDieR Checklist was used alongside the TIDieR Guide [67], which provides detailed instructions for using the checklist and explains each item comprehensively.

For our second objective, we systematically categorized the interventions using the Behavior Change Wheel (BCW) [64], identifying and classifying the intervention functions and the sources of behavior change to better understand how these interventions are intended to influence behavior. We categorized the intervention functions and behavior sources using the BCW as a categorizing framework. According to Michie et al. [64], intervention functions are “broad categories of means by which an intervention can change behavior” (p. 109). The categorization of intervention functions for each retrieved item was based on Michie's definitions and examples. Michie et al. [64] has identified nine functions: education, persuasion, incentivization, coercion, training, restriction, environmental restructuring, modelling, and enablement. Still using the BCW model, the source of behavior change was inferred based on the COM‐B (capacity‐opportunity‐motivation‐behavior) model [64]. The core principle of this model is that for any behavior to occur, there must be the “capacity” to perform it, the “opportunity” for it to happen, and sufficient “motivation.” Referring to COM‐B will help us evaluate how existing interventions address the three components of behavior change. Figure 1 outlines the definitions of each intervention function and the sources of behaviors that was used to categorize and describe the model of change for each intervention.

**FIGURE 1:**
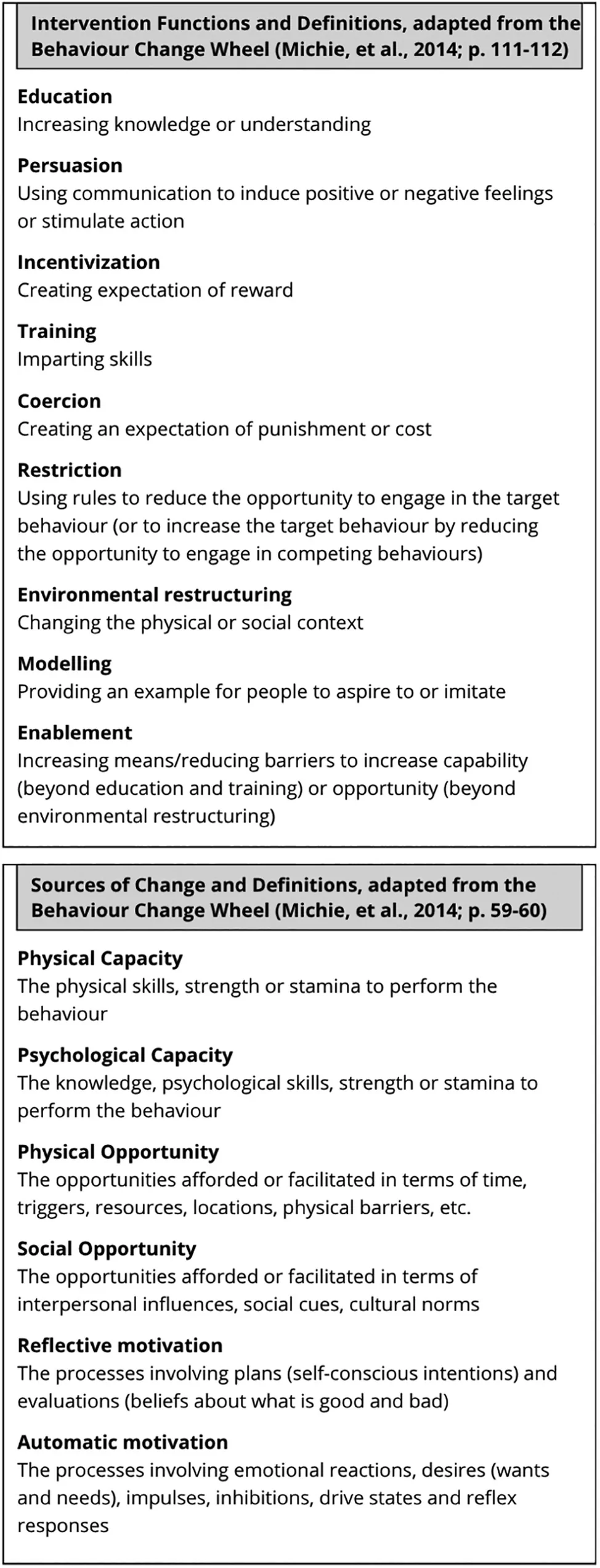
Definitions of intervention functions and sources of behavior change, adapted from the Behavior Change Wheel (Michie et al. [64]).

The ORBIT (Obesity‐Related Behavioral Intervention Trials) model was used as a framework for classifying the stage of intervention development [69]. Studies were classified based on their stated aims, design characteristics, and primary purpose rather than solely on authors' labels. Specifically, studies focused on intervention acceptability, feasibility, or preliminary optimization were classified as ORBIT Phase IIb (feasibility/optimization), whereas studies primarily designed to evaluate preliminary efficacy with a comparison condition were classified as Phase III.

After screening and final inclusion, all retained articles underwent a final verification review to confirm bibliographic accuracy and to ensure that no included publications had been retracted or issued major editorial notices.

## 3 Results

Our search identified a total of 6170 items from the electronic databases (including a search update yielding 155 records). Among them, 2894 duplicate records were identified and removed, leaving 3276 items eligible for screening. After the first analysis by titles and abstracts, 2784 items were excluded. A total of 492 items were assessed for full‐text eligibility, and 469 items did not meet the inclusion criteria (see Figure 2 for a compilation of reasons for exclusion, in accordance with the PRISMA‐ScR statement). A total of 22 items initially met the eligibility criteria and were included in this scoping review. Following the updated search, one additional record met the inclusion criteria and was added, resulting in a total of 23 included items. Figure 2 presents the PRISMA flow diagram from this scoping review.

**FIGURE 2:**
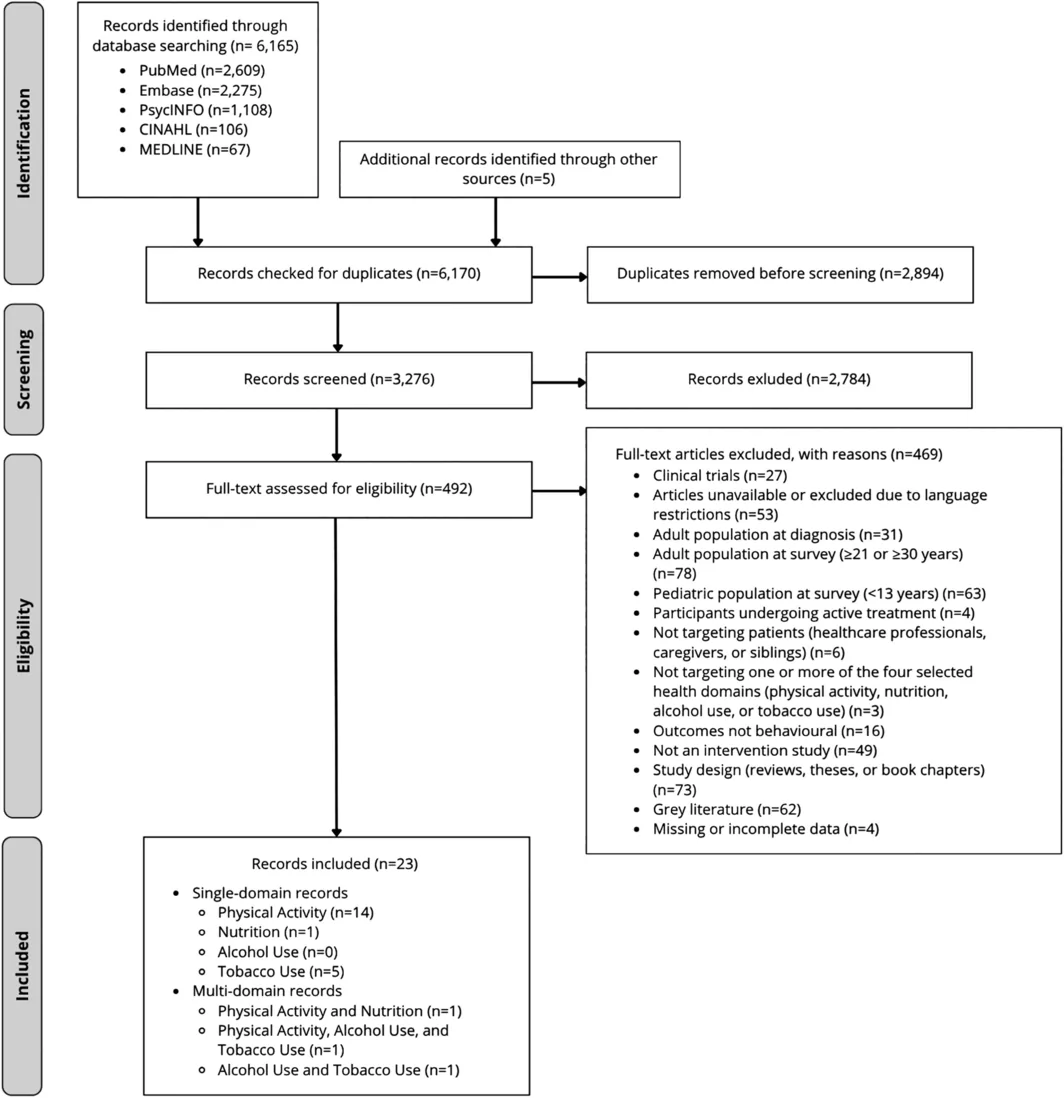
PRISMA‐ScR flowchart of the literature search and study selection.

Multiple reports describing the same intervention (e.g., protocol publications, implementation reports, and outcome papers) were linked and coded under a single intervention record. Summary characteristics were therefore reported based on the 17 distinct interventions, while the 23 included items represented the total number of publications contributing descriptive information. Results describing the characteristics of interventions were calculated using the 17 distinct interventions as the denominator, whereas results describing the characteristics or frequency of publications were calculated using the 23 included publications as the denominator.

Among the 23 items [62, 66, 70, 71, 72, 73, 74, 75, 76, 77, 78, 79, 80, 81, 82, 83, 84, 85, 86, 87, 88, 89, 90], three were research protocols [62, 70, 86]. The items were published from 2005 to 2025, and they were conducted in the USA (14, 61%) [66, 70, 71, 72, 73, 74, 75, 76, 77, 78, 79, 82, 84, 90], in Switzerland (3, 13%) [86, 87, 88], Germany (2, 8.6%) [85, 89], Canada (2, 8.6%) [80, 81], and Australia (2, 8.6%) [62, 83]. Across the included empirical publications, 3847 participants were reported, with a total of 1831 (48%) women, 1939 (50%) men, and 77 (2%) participants whose sex or gender was reported as other or was not specified. Because some interventions were represented by multiple records, these figures may include overlapping participant samples and therefore reflect the total number of participants reported across publications rather than the number of unique participants.

Sample sizes ranged from 10 to 796 participants. The ages of participants at diagnosis and at the time of the survey were not reported systematically in the published items. When available, participant ages are individually listed for each study. The variability and inconsistent reporting of key participant characteristics, including age at diagnosis, age at intervention, and treatment history, reflect the heterogeneity of the current evidence base and highlight the need for more standardized reporting practices to improve the comparability and interpretation of future research.

Twelve of the twenty empirical studies [66, 73, 74, 77, 78, 81, 82, 83, 84, 85, 88, 90] were feasibility studies or stated feasibility in the objectives, corresponding to Phase IIb of the ORBIT model. Eight of the 20 studies [71, 72, 75, 76, 79, 80, 87, 89] were reported as efficacy trials, corresponding to Phase III of the ORBIT model [69]. Table 1 presents descriptions of the 23 extracted records.

**TABLE 1:** Summary of study characteristics and key findings.

Authors (country)	Name of intervention (if provided)/*intervention description*	Participant characteristics, age in years	Study design	Aim of the article	Outcomes [measures] (IMP = implicitly stated, EXP = explicitly stated)	Assessment	Main finding	Conclusion
Physical activity								
Beight et al. (2024) (USA)	StepByStep *Mobile health and social media physical activity intervention*	*N* = 383 *Gender* F = 167; M = 147 O = 12; DNA = 57 *Age at diagnosis*: Mo: 15.2 ± 2.22 (R: 1.9–20.3) *Age at time of study*: Mo: 17.9 ± 1.7 (R: 15.0–20.9) *Type of cancer*: Acute lymphoblastic leukemia, other leukemia, lymphoma, central nervous system tumor, sarcoma, and other solid tumor	Two‐arm prospective RCT	Evaluate the efficacy of a 48‐week multi‐component mHealth and social media intervention in MVPA.	**Primary outcome (EXP)**: Minutes of MVPA per day [accelerometer data] **Secondary outcome (EXP)**: Change in light PA and sedentary behavior [accelerometer data] Cardiometabolic health [blood sample test and metabolic data] Blood biomarkers [blood sample test] HRQoL [Pediatric Quality of Life Inventory 4.0 Generic Core Scales (global physical, social functioning scales) and 4.0 Multidimensional Fatigue Scale]	T0: baseline T1: after intensive phase of intervention (Week 24) T2: after maintenance phase of intervention (Week 48)	Only demographic and clinical characteristics of enrolled participants were collected (ongoing study)	Only demographic and clinical characteristics of enrolled participants were collected (ongoing study)
Berg et al. (2014)^a^ (USA)	*Online interventions targeting health promotion*	*N* = 24 *Gender*/*sex* ^b^ F = 17; M = 7 *Time since diagnosis*: M = 10.71 (SD = 6.22) *Age at time of study*: M = 23.38 (SD = 3.91) *Type of cancer*: Hodgkin's lymphoma, acute lymphoblastic leukemia; acute myelogenous leukemia, osteosarcoma; thyroid cancer, glioblastoma; and Wilm's tumor.	One‐arm pretest post‐test pilot trail	Examine feasibility and acceptability of a web‐based healthy lifestyle intervention [PA, alcohol, tobacco] for young adult childhood cancer survivors.	**Primary outcomes (IMP)**: Feasibility and acceptability of intervention **Secondary outcomes (IMP)** Activity level Alcohol consumption Tobacco consumption Psychosocial functions	T0: baseline T1: end‐of‐tx (Week 6) T2: follow‐up (Week 12)	Feasible (high recruitment/retention), acceptable (85.7% satisfied, and strong receptivity) No significant improvements documented from pre‐ to post‐test for either PA level, alcohol consumption or tobacco consumption	Although no significant behavior changes were documented from pre‐ to post‐test, the study demonstrated feasibility and acceptability, providing insights to guide future intervention development.
Devine et al. (2020) (USA)	FitSurvivors *Technology‐enhanced group‐based fitness intervention*	*N* = 49 (Int. group; *n* = 25) *Gender/sex* F = 24; M = 25 *Age at diagnosis*: Int. group; M = 7.36 (SD = 5.5) Waitlist; M = 8.54 (SD = 6.1) *Age at time of study*: Int. group; M = 18.76 (SD = 3.9) Waitlist; M = 18.25 (SD = 3.6) *Type of cancer*: Blood cancer, brain tumor and solid tumor	RCT, with waitlist control	Evaluating the feasibility and acceptability of FitSurvivor, and examining the effects of physical activity, muscular strength, cardiorespiratory fitness, fatigue, and HRQoL	**Primary outcomes (EXP)**: Feasibility and acceptability [enrollment, retention, attendance, app engagement, satisfaction] **Secondary outcomes (EXP)**: Physical activity and sedentary [daily MVPA, with accelerometer and self‐report] behavior, muscular strength, cardiorespiratory fitness (including VO2max, BMI), fatigue, and HRQoL (PedsQL Generic Core)	T0: baseline T1: follow‐up 2 months post‐randomization T2: follow‐up 3 months post‐randomization T3: follow‐up 6 months post‐randomization T4: follow‐up 9 months post‐randomization	Acceptable feasibility/acceptability (limited by geographic barriers, competing demands). No significant group*time effects for PA/sedentary behavior Intervention group improved lower body strength significantly vs. WL, with small nonsignificant gains in other fitness outcomes. No significant differences in HRQoL or fatigue	Showed promise for engaging participants, but reach was limited by geographic barriers and competing demands of AYA survivors.
Faith et al. (2024)^a^ (USA)	ENHANCE; Empowering New Habits and Nutrition After the Cancer Experience *Telehealth individually tailored healthy lifestyle intervention*	*N* = 58 (Int. group; *n* = 35) *Gender* F = 34; M = 16; O = 2 Unreported = 6 *Age at diagnosis*: Int. group; M = 7.79 (SD = 5.34) Contr. group; M = 10.30 (SD = 5.02) *Age at time of study*: Int. group; M = 18.80 (SD = 2.61) Contr. group; M = 18.68 (SD = 2.46) *Type of cancer*: Hematological cancer, CNS tumor, and non‐CNS tumor	Pilot RCT, two‐group (intervention, control)	To evaluate feasibility and acceptability of a telehealth intervention (ENHANCE) supporting AYACS' nutrition and physical activity	**Outcomes of article (EXP)**: Feasibility and acceptability **Intervention outcomes (IMP)**: Nutrition behavior Physical activity behavior Healthy lifestyle efficacy Internalizing symptoms Self‐esteem and weight control	T1: baseline Int. Group T2: at completion T3: 10–14 weeks after completion T4: 22–26 weeks after completion Contr. Group; T2: 10–14 weeks after education T3: 22–26 weeks after education T4: 34–28 weeks after education	High feasibility (96.7% completed all sessions); strong fidelity (full content delivered in all but 2 sessions). Acceptability supported. Participants valued interventionist support, though some suggested tailoring to baseline knowledge.	ENHANCE was feasible and highly acceptable; most AYACS participated without a care partner, supporting feasibility of a future larger RCT.
Ha et al. (2025) (Australia)	Making Movers *Home‐based health behavior educational program*	*RESEARCH PROTOCOL*	Hybrid effectiveness‐implementation pre‐post trial	Evaluate the potential effectiveness of Making Moves on CCS physical activity self‐efficacy and other health outcomes	**Primary outcome (EXP)**: Physical activity self‐efficacy [Physical Activity Self‐Efficacy Scale (PASES)] **Secondary outcomes (EXP)**: Objective PA level (accelerometer) Subjective PA level [International Physical Activity Questionnaire for Adolescents], Symptoms of depression [12‐item Centre for Epidemiological Studies Depression Scale], Readiness to exercise [using transtheoretical model], Perceived enjoyment of exercise [PACES], HR‐QoL [EQ‐5d‐Y‐5 L], Aerobic fitness [6‐min walk test], Muscular strength [sit‐to‐stand test]	T0: baseline T1: optional check‐in session at Week 2 T2: post‐intervention T3: booster session, 3 months after completion T4: 6‐month follow‐up		
Keats et al. (2008) (Canada)	*Theory‐based physical activity program*	*N* = 10 *Gender* F = 8; M = 2 *Time since diagnosis*: M = 5.21 *Age at time of study*: M = 16.20 (SD = 1.62) R (14–18) *Type of cancer*: Lymphoma, leukemia, central nervous system tumor, and germ cell tumor	Single group prospective study	Examine the feasibility of a theoretically based PA intervention in adolescent survivors of cancer	**Primary outcomes (EXP)**: Feasibility (recruitment, adherence, and retention) **Intervention outcomes (EXP)**: Total PA [Godin Leisure‐Time Exercise Questionnaire], physical fitness [Fitness Testing—Fitnessgram—Cooper Institute], overall QoL [Pediatric Quality of Life Inventory (PedsQL)], fatigue [Teen Pediatric Quality of Life Multidimensional Fatigue Scale], social, emotional and physical well‐being	T0: baseline T1: mid‐intervention (after 8 weeks) T2: post‐intervention (after 16 weeks) T3: follow‐up (3 months post‐intervention) T4: follow‐up (12 months post intervention)	Attendance over the 16‐week intervention averaging 81.5%. Improvements in total PA, physical fitness, and QOL were noted across the intervention. Follow‐up data revealed that participants failed to maintain their postintervention PA levels at both the 3 and 12‐month follow‐up assessments.	Despite initial improvements in PA, fitness, and QOL, participants did not maintain PA gains at 3‐ or 12‐month follow‐up.
Keats et al. (2009) (Canada)	*Theory‐based physical activity program*	*N* = 10 *Gender/sex* F = 8; M = 2 *Time since diagnosis*: M = 5.21 *Age at time of study*: M = 16.20 (SD = 1.62) R (14–18) *Type of cancer*: Lymphoma, leukemia, central nervous system tumor, and germ cell tumor	Prospective pilot study	Examine the impact of the intervention on the key theoretical constructs within the TPB Examine the predictive utility of the TPB in understanding PA intention and behavior within a prospective design.	**Primary outcomes (IMP)**: Planned behavior concepts (affective attitude, instrumental attitude, subjective norm, controllability, self‐efficacy, intention) **Secondary outcomes (IMP)**: PA leisure frequency (Godin Leisure‐Time Exercise Questionnaire [GLTEQ])	T0: baseline T1: mid‐intervention (after 8 weeks) T2: post‐intervention (after 16 weeks) T3: follow‐up (3 months post‐intervention) T4: follow‐up (12 months post intervention)	Small but meaningful impact on behavioral intentions; effects were limited by ceiling effects from high baseline attitudes, norms, and perceived control.	Produced small but meaningful changes in TPB constructs and provided preliminary support for the TPB's predictive utility in explaining PA intentions and behavior among pediatric cancer survivors.
Le et al. (2017) (USA)	*Home‐based activity tracker intervention*	*N* = 19 *Gender* F = 14; M = 5 *Time since diagnosis*: M = 14.4 (SD = 8.0) R (1.7–28.9) *Age at time of study*: M = 24.3 (SD = 5.8) R (15–35) *Type of cancer*: Leukemia, lymphoma, sarcoma, CNS tumor, and Wilms tumor	Pilot cohort study	Evaluation of the feasibility of a home‐based exercise intervention using a motivational activity tracker in pediatric cancer survivors	**Primary outcomes (IMP)**: Feasibility, impact on activity level [activity tracker] and physical fitness [cardiopulmonary testing] **Secondary outcomes (IMP)**: Barriers, preferences and beliefs regarding PA, MVPA	T0: baseline: pre‐intervention T1: daily measure (for 6 months) T3: Follow‐up post‐intervention	79% retention rate. MVPA increased modestly but not significantly. Self‐reported behaviors showed no change All participants would recommend and continue using the Fitbit.	The program was feasible and did increase the level of physical activity, although not statistically significant
Long et al. (2018) (Australia)	*Exercise intervention program*	*N* = 13 *Sex* F = 7; M = 6 *Age at diagnosis*: M = 3 R (0–10) *Age at time of study*: *M = 19 R* (16–23) *Type of cancer*: Brain tumor, leukemia, and other types of cancer	Cohort study	Assessed the feasibility and effectiveness of a 24‐week exercise intervention program in pediatric oncology survivors	**Primary outcomes (EXP)**: CVD risk [via endothelial function] **Secondary outcomes (EXP)**: Aerobic capacity, resting heart rate, blood pression, physical activity levels [accelerometer], body composition, strength, and endurance	T0: baseline T1: after 24 weeks of regular care T2: after 24 weeks of exercise	Physical activity remained below recommendations (6.6 ± 4.0 to 13.9 ± 9.5 min/day) Sedentary breaks significantly increased following the exercise program. Improvement in vascular function and risk of cardiovascular event.	Implementation of the intervention is feasible but challenging.
Mendoza et al. (2017) (USA)	*Fitbit and Facebook mHealth promoting intervention*	*N* = 59 (Int. group *n* = 29) *Gender* F = 35; M = 24 *Time since diagnosis*: M = 10.3 *Age at time of study*: M = 16.6 (SD = 1.5) *Type of cancer*: Leukemia (ALL, AML), bone (includes Ewing, osteosarcomas), central nervous system, lymphoma (includes Hodgkin and other types), and other solid tumors, other	Two‐arm pilot RCT	Evaluated a Fitbit and Facebook intervention program compared to a control condition of usual care	**Primary outcomes (EXP)**: Feasibility and acceptability **Secondary outcomes (EXP)**: Physical activity [activity tracker], QoL [Pediatric Quality of life Inventory], functioning [Generic Core], engagement [Facebook group], Cancer‐related symptoms [Cancer Model Scales], need satisfaction [Psychological Need Satisfaction Exercise Scale], behavioral regulation [Behavioral Regulation Exercise], enjoyment [Physical Activity Enjoyment Scale]	T1: baseline T2: during Weeks 8–10 of the intervention	All feasibility criteria were achieved (*n* = 60 recruited; 71.5% Fitbit wear; 90–100% questionnaire completion). No significant group differences were observed in PA or sedentary time. Modest effects for some QoL and motivation subscales. Qualitative data supported acceptability.	The intervention appears to be both feasible and acceptable
Miller et al. (2021) (USA)	GECCOS trial; Green Exercise for Childhood Cancer Survivors *Indoor and outdoor exercise training program*	*N* = 19 *Sex* F = 10; M = 9 *Age at diagnosis*: Not reported *Age at time of study*: M = 19.7 R (13.3–27.6) *Type of cancer*: Acute lymphoblastic leukemia, acute myeloid leukemia, central nervous system, and other (including Hodgkins lymphoma, Wilms tumor, Langerhans cell histiocytosis; aplastic anemia; posttransplant lymphoproliferative disorder; rhabdomyosarcoma embryonal; Ewings sarcoma and soft tissue sarcoma)	Randomized cross‐over pilot trial	Assessed the feasibility of recruiting survivors from a long‐term follow‐up clinic to an exercise group and measure whether outdoor or indoor exercise sessions better supported exercise motivation and behaviors in survivors of cancer	**Primary outcomes (IMP)**: Weekly MVPA [METs] **Secondary outcomes (IMP)**: Perceived autonomy, competence and relatedness [Psychological Need Satisfaction in Exercises; PNSE] Exercise Motivation [Behavior regulation in exercice questionnaire; BREQ‐2], Fatigue [Fatigue Scale‐adolescent; FSA]	T0: at baseline T1: after Week 1 T2: after Week 4	Only the extrinsic motivation subscale of the BREQ‐2 showed significant change after indoor versus outdoor exercise. Participants worked out at a significantly higher intensity during outdoor sessions compared to indoor sessions.	Showed that recruiting survivors to community‐based exercise groups is feasible, outdoor activity promotes greater exercise intensity than indoor activity, and the findings offer guidance for future programs.
Rueegg et al. (2017) (Switzerland)	SURFit *Supervised physical activity program*	*RESEARCH PROTOCOL*	RCT	To evaluate the effect of a partially supervised and personalized PA program	**Primary outcomes (EXP)**: Composite CVD risk scores [battery of blood and physiological test] **Secondary outcomes (EXP)**: CVD risk factors, glycaemic control, bone health, body composition, physical activity [pedometer and accelerometer, Seven‐day physical activity recall; PAR], physical fitness, QoL, mental health, fatigue and adverse events	T0: baseline T3: after 3 months T6: after 6 months T12: after 12 months		
Rueegg et al. (2023) (Switzerland)	SURFit *Supervised physical activity program*	*N* = 151 (Int. group *n* = 76) *Sex* F = 66; M = 85 *Age at diagnosis*: Int. group; M = 7.6 (SD = 5.1) Contr. group; M = 7.3 (SD = 4.6) *Age at time of study*: Int. group; M = 31.5 (SD = 8.3) Contr. group; M = 29.3 (SD = 8.7) *Type of cancer*: Leukemia, lymphoma; CNS tumors; bone tumors/soft tissue sarcomas; and other diagnoses	Two‐arm parallel RCT	Investigated the effect of a partially supervised, personalized, 1‐year PA intervention	**Primary outcomes (EXP)**: Change in composite CVD risk score **Secondary outcomes (EXP)**: Metabolic and Glycemic Control (i.e., fasting glucose, insulin resistance), cardiovascular health (blood pressure, cholesterol, triglycerides), anthropometrics and body composition (waist circumference, body mass index, body fat mass), physical fitness (peak performance, strength, endurance) physical activity and sedentary behavior (total physical activity, MVPA, [accelerometer]sedentary time, number of steps per day [pedometer])	T0: baseline T6: after 6 months T12: after 12 months	Significant impact on CVD risk score in the intervention group. No effect on PA parameters measured by the accelerometer and pedometers	No effect on physical activity levels and habits. Significant impact on CVD risk score
Jung et al. (2023) (Switzerland)	SURFit *Supervised physical activity program*	*N* = 144 *Sex* F = 62; M = 82 *Age at diagnosis*: Int. group; M = 7.5 (SD = 5.1) Contr. group; M = 7.5 (SD = 4.7) *Age at time of study*: Int. group; M = 31.6 (SD = 8.5) Contr. group; M = 29.3 (SD = 8.8) *Type of cancer*: Leukemia, lymphoma, central nervous system tumor, and other tumors	Two‐arm parallel RCT	Described and identified the predictors of exercise adherence and contamination of the intervention and control group with an RCT	**Primary outcomes (EXP)**: Adherence and contamination	T0: baseline T6: after 6 months T12: after 12 months	Adherence to intervention was 48% in the intervention group and 17% in the control group. Predictors for PA adherence: female sex, higher physical and mental quality of life, week into the intervention. No significant predictors for contamination were found for controls.	Adherence remained challenging in both groups, with clear differences in physical activity behavior observed between adherent and non‐adherent participants.
Salchow et al. (2021) (Germany)	MAYA trial; Motivate AYA *Structured counseling‐based intervention to improve physical activity*	*N* = 89 (Int. group *n* = 45) *Sex* F = 49; M = 40 *Age at diagnosis*: M = 15.9 (SD = 9.0) *Age at time of study*: M = 24.1 (SD = 6.3) *Type of cancer*: Solid tumors, lymphoma, and leukemia	Two‐arm RCT	Explored whether a structured counseling‐based intervention increases vigorous activity behaviors in AYA pediatric cancer survivors	**Primary outcomes (EXP)**: Rate of vigorous activity [METs] **Secondary outcomes (EXP)**: Amount and intensity of PA behavior [International Physical activity Questionnaire; IPAQ], overall QoL [European Organisation for Research and Treatment of Cancer Quality of Life Questionnaire Core 30 (EORTC‐QL‐C30)]	T0: baseline T1: post‐intervention at Week 12 T2: follow‐up at Week 52	The intervention did not significantly increase vigorous activity compared to controls. Follow‐up showed sustained favorable trends in the intervention group with higher vigorous activity, reduced sitting time, and increased total MET score, alongside a decrease in moderate PA at 12 weeks.	The intervention did not improve vigorous activity in AYA cancer survivors
Williamson‐Lewis et al. (2023) (USA)	*Fitbit fitness intervention*	*N* = 44 *Sex* F = 23; M = 21 *Age at diagnosis*: M = 5.8 (SD = 3.3) *Age at time of study*: M = 15.1 (SD = 1.2) *Type of cancer*: Leukemia, lymphoma, solid tumor, and aplastic anemia	Prospective study	Characterized the feasibility of adolescent‐aged survivors of childhood cancer using a wearable activity tracker, and identify those who used it in the absence of incentives, reminders or interventions	**Primary outcomes (IMP)**: Feasibility and adherence **Secondary outcomes (IMP)**: Activity tracker use Device satisfaction and acceptability	T0: baseline T1: follow‐up after 12 months (±3 months)	Survivors found Fitbits useful for self‐monitoring and motivating activity, but challenges with device use, lack of incentives, and absence of structured support led to poor adherence, with consistent use dropping below one‐third in the first 12 weeks and nearly disappearing by 1 year.	Results suggest that wearable activity trackers must be combined with other behavior change strategies and reminders to wear, charge, and sync to be feasible and maximize engagement.
Nutrition								
Faith et al. (2024)^a^ (USA)	ENHANCE; Empowering New Habits and Nutrition After the Cancer Experience *Telehealth individually tailored healthy lifestyle intervention*	*N* = 58 (Int. group; *n* = 36) *Gender* F = 34; M = 16; O = 2 Unreported = 6 *Age at diagnosis*: Int. group; M = 7.79 (SD = 5.34) Contr. group; M = 10.30 (SD = 5.02) *Age at time of study*: Int. group; M = 18.80 (SD = 2.61) Contr. group; M = 18.68 (SD = 2.46) *Type of cancer*: Hematological cancer, CNS tumor, and non‐CNS tumor	Pilot RCT, two‐group (intervention, control)	To evaluate feasibility and acceptability of a telehealth intervention (ENHANCE) supporting AYACS' nutrition and physical activity	**Outcomes of article (EXP)**: Feasibility and acceptability **Intervention outcomes (EXP)**: Nutrition behavior Physical activity behavior Healthy lifestyle efficacy Internalizing symptoms Self‐esteem and weight control	T1: baseline Int. Group T2: at completion T3: 10–14 weeks after completion T4: 22–26 weeks after completion Contr. Group; T2: 10–14 weeks after education T3: 22–26 weeks after education T4: 34–28 weeks after education	High feasibility (96.7% completed all sessions); strong fidelity (full content delivered in all but 2 sessions). Acceptability supported. Participants valued interventionist support, though some suggested tailoring to baseline knowledge.	ENHANCE was feasible and highly acceptable; most AYACS participated without a care partner, supporting feasibility of a future larger RCT.
Quidde et al. (2016) (Germany)	INAYA; Improved Nutrition for AYA *Intensified individual nutrition counseling*	*N = 23* *Gender*/*sex* ^b^ F = 11, M = 12 *Age at diagnosis*: Md = 16.0 (R: 10.0–17.0) *Age at time of study*: Md = 20 (R: 19–23) *Type of cancer*: Sarcoma, carcinoma, blastoma, Hodgkin's lymphoma, and leukemia	Pilot study	Feasibility and impact of an intensified nutrition counseling in the AYA childhood cancer survivors' populations	**Primary outcomes (EXP)**: Nutritional Behaviors [Healthy Eating Index—European Prospective Investigation into Cancer and Nutrition (HEI‐EPIC)] **Secondary outcomes (EXP)**: Waist‐hip ratio [metrics], Body mass index [metrics], HRQoL [EORTC QLQ‐C30] Blood Pressure [metrics], and other secondary parameters	T0: baseline T1: Week 2 T2: Week 6 T3: Week 12	Improvement of 20 points in HEI EPIC score in 52.2% (*n* = 12) of AYA. No change was seen in HRQoL, waist‐hip ratio or blood‐pressure	An intensified, individualized nutrition counseling is feasible in this patient group. Associated with notable improvements in nutritional behavior
Alcohol consumption								
Berg et al. (2014)^a^ (USA)	*Online interventions targeting health promotion*	*N* = 24 *Gender*/*sex* ^b^ F = 17; M = 7 *Time since diagnosis*: M = 10.71 (SD = 6.22) *Age at time of study*: M = 23.38 (SD = 3.91) *Type of cancer*: Hodgkin's lymphoma, acute lymphoblastic leukemia; acute myelogenous leukemia, osteosarcoma; thyroid cancer, glioblastoma; and Wilms' tumor.	One‐arm pretest post‐test pilot trail	Examine feasibility and acceptability of a web‐based healthy lifestyle intervention [PA, alcohol, tobacco] for young adult childhood cancer survivors.	**Primary outcomes (IMP)**: Feasibility and acceptability of intervention **Secondary outcomes (IMP)** Activity level Alcohol consumption Tobacco consumption Psychosocial functions	T0: baseline T1: end‐of‐tx (Week 6) T2: follow‐up (Week 12)	Feasible (high recruitment/retention), acceptable (85.7% satisfied, strong receptivity). No significant improvements documented from pre‐ to post‐test for either PA level, alcohol consumption or tobacco consumption.	Although no significant behavior changes were documented from pre‐ to post‐test, the study demonstrated feasibility and acceptability, providing insights to guide future intervention development.
Hollen et al. (2013)^a^ (USA)	Decision KEYS for Balancing Choices *Substance use decision aid*	*N* = 243 *Sex* F = 128; M = 115 *Age at diagnosis*: M = 5.1 R (0–14) *Age at time of study*: M = 16.3 R (1.6) *Type of cancer*: Acute lymphoblastic leukemia, acute myelogenous leukemia, Hodgkin lymphoma, non‐Hodgkin lymphoma, Sarcoma, Embryonal, brain tumors, and other	Prospective RCT	Tested a decision aid for adolescent survivors of childhood cancer that is aimed at difficult decisions related to engaging in substance use risk behaviors.	**Primary outcomes (IMP)**: Decision making, Risk motivation, Substance use risk behaviors, planned contrasts, evaluation of intervention	T0: baseline T1: Mid‐point intervention (at 6 months) T2: End of intervention (12 months)	The intervention was well received by adolescent survivors and showed short‐term benefits in reducing risk motivation, particularly for alcohol and drug‐related behaviors, though no sustained effects were observed on decision‐making outcomes at 6 or 12 months.	Demonstrated only modest effects; while teens reported improved perceptions of their decision‐making ability after engaging with the program, findings highlight the need for a more diagnostic baseline measure and a more intensive intervention in the final 6 months.
Tobacco consumption								
Berg et al. (2014)^a^ (USA)	*Online interventions targeting health promotion*	*N* = 24 *Gender*/*sex* ^b^ F = 17; M = 7 *Time since diagnosis*: M = 10.71 (SD = 6.22) *Age at time of study*: M = 23.38 (SD = 3.91) *Type of cancer*: Hodgkin's lymphoma, acute lymphoblastic leukemia; acute myelogenous leukemia, osteosarcoma; thyroid cancer, glioblastoma; and Wilms' tumor.	One‐arm pretest post‐test pilot trail	Examine feasibility and acceptability of a web‐based healthy lifestyle intervention [PA, alcohol, tobacco] for young adult childhood cancer survivors.	**Primary outcomes (IMP)**: Feasibility and acceptability of intervention **Secondary outcomes (IMP)** Activity level Alcohol consumption Tobacco consumption Psychosocial functions	T0: baseline T1: end‐of‐tx (Week 6) T2: follow‐up (Week 12)	Feasible (high recruitment/retention), acceptable (85.7% satisfied, strong receptivity) No significant improvements documented from pre‐ to post‐test for either PA level, alcohol consumption or tobacco consumption	Although no significant behavior changes were documented from pre‐ to post‐test, the study demonstrated feasibility and acceptability, providing insights to guide future intervention development.
Asfar et al. (2010) (USA)	St. Jude Cancer Survivors Quit Line *Tobacco quit line*	*RESEARCH PROTOCOL*	Two‐arm RCT	To evaluate the long‐term efficacy of a counselor initiated quit line, in two different intervention groups	**Primary outcomes (EXP)**: Abstinence **Secondary outcomes (EXP)**: Adherence and cessation outcomes, dose of NRT treatment	T0: baseline T1: end of intervention (Week 8) T2: 12‐month follow‐up		
Klesges et al. (2015) (USA)	St. Jude Cancer Survivors Quit Line *Tobacco quit line*	*N* = 519 *Sex* F = 234; M = 285 *Age at diagnosis*: Not provided *Age at time of study*: Not provided *Type of cancer*: Not provided	Parallel‐group RCT	Determined the efficacy of a tobacco quit line for adult tobacco used who survived childhood cancer, and determined if the quit rate would be significantly different in the two conditions	**Primary outcomes (EXP)**: Abstinence **Secondary outcomes (EXP)**: Self‐reported abstinences and continuous quit rate	T0: baseline T1: end of intervention (Week 8) T2: 12‐month follow‐up	Despite high self‐reported abstinence, testing revealed that 80% of CCS who claimed abstinence were still using tobacco, resulting in true cessation rates below 2%. While the Proactive +4 weeks condition showed significantly higher short‐term self‐reported abstinence than the Reactive +2 weeks condition, biochemically adjusted rates were very low, and no significant differences persisted at 12 months.	Neither intervention groups significantly impacted long‐term smoking cessation rates.
Emmons et al. (2005) (USA)	Partnership for Health (PFH) *Telephone‐based peer‐delivery smoking cessation intervention*	*N* = 796 *Sex* F = 374; M = 422 *Age at diagnosis*: R (0–3); *n* = 151 R (4–9); *n* = 231 R (10–14); *n* = 223 > 15; *n* = 191 *Age at time of study*: M = 31.0 (SD = 6.66) *Type of cancer*: Leukemia, Hodgkin's disease, CNS malignancy, non‐Hodgkin's lymphoma, Bone cancer, soft tissue sarcoma, kidney cancer, and neuroblastoma	Two‐group RCT	Evaluated the smoking status and smoking behavior outcomes of childhood cancer survivors following a counseling and peer‐based intervention	**Primary outcomes (EXP)**: Smoking status (7‐day point) **Secondary outcomes (EXP)**: Smoking behaviors (number of quit attempts, smoking rate, nicotine dependence, NRT used), cost of intervention provision	T0: baseline T1: 8‐month follow‐up T2: 12‐month follow‐up	The proactive counseling intervention significantly increased quit rates compared to self‐help, with benefits sustained at 12 months. Baseline self‐efficacy strongly predicted cessation, with high self‐efficacy survivors four times more likely to quit. Higher quit rates observed among participants receiving more counseling calls. The intervention was substantially more costly than self‐help.	The intervention did not increase quit attempts but improved their success, with the PFH counseling program doubling smoking cessation rates compared to self‐help.
Emmons et al. (2009) (USA)	Partnership for Health (PFH) *Telephone‐based peer‐delivery smoking cessation intervention*	*N* = 796 *Sex* F = 371; M = 425 *Age at diagnosis*: Not reported *Age at time of study*: M = 31 (SD = 6.5–6.8 depending on intervention group) *Type of cancer*: Bone/STS/Wilms', CNS, Leukemia/Lymphoma	Two‐group RCT	Determined whether the proactive counseling group or the self‐help group wielded more results [implicit]	**Primary outcomes (EXP)**: Smoking status **Secondary outcomes (EXP)**: Self‐efficacy, readiness to quit, depressed mood.	T0: baseline T1: 8‐month follow‐up T2: 12‐month follow‐up T3: Long‐term follow‐up	At long‐term follow‐up, proactive counseling produced higher quit rates than self‐help, with outcomes strongly influenced by nicotine dependence, education, self‐efficacy, and greater use of nicotine replacement therapy, the latter mediating the intervention's effect.	Cessation rates continue to be significantly higher among participants in the PC condition versus SH, although the differences were not large.
Park et al. (2006) (USA)	Partnership for Health (PFH) *Telephone‐based peer‐delivery smoking cessation intervention*	*N* = 398 *Sex* F = 189; M = 209 *Age at diagnosis*: M = 9.7 (SD = 6.0) *Age at time of study*: M = 30.9 (SD = 6.5) *Type of cancer*: Bone cancer, CNS, Hodgkin's disease, Leukemia, Neuroblastoma, Non‐Hodgkin's lymphoma, Soft tissue sarcoma, Wilm's	RCT	Described a survivor‐targeted intervention, evaluate its implementation, and examine how participant characteristics and engagement related to smoking outcomes.	**Primary outcomes (EXP)**: Smoking status **Secondary outcomes (EXP)**: Quit attempts, motivation to quit smoking, stages of readiness	T0: baseline T1: 8‐month follow‐up T2: 12‐month follow‐up	A brief, peer‐delivered telephone counseling intervention was feasible and engaging for most survivors, with greater involvement linked to demographic and health factors. Counseling dose significantly predicted cessation, as those receiving the maximum number of calls were far more likely to quit at 12 months, while NRT use was marginally associated with quit efforts.	Shows promise for supporting smoking cessation among adult childhood cancer survivors, with process evaluation findings on call delivery providing guidance for future program design.
Hollen et al. (2013)^a^ (USA)	Decision KEYS for Balancing Choices *Substance use decision aid*	*N* = 243 *Sex* F = 128; M = 115 *Age at diagnosis*: M = 5.1 R (0–14) *Age at time of study*: M = 16.3 R (1.6) *Type of cancer*: Acute lymphoblastic leukemia, acute myelogenous leukemia, Hodgkin lymphoma, non‐Hodgkin lymphoma, sarcoma, embryonal, brain tumors, and other	Prospective RCT	Tested a decision aid for adolescent survivors of childhood cancer that is aimed at difficult decisions related to engaging in substance use risk behaviors.	**Primary outcomes (IMP)**: Decision making, Risk motivation, Substance use risk behaviors, planned contrasts, evaluation of intervention	T0: baseline T1: mid‐point intervention (at 6 months) T3: end of intervention (12 months)	The intervention was well received by adolescent survivors and showed short‐term benefits in reducing risk motivation, particularly for alcohol and drug‐related behaviors, though no sustained effects were observed on decision‐making outcomes at 6 or 12 months.	Demonstrated only modest effects; while teens reported improved perceptions of their decision‐making ability after engaging with the program, findings highlight the need for a more diagnostic baseline measure and a more intensive intervention in the final 6 months.

Abbreviations: CVD, cardiovascular disease(s); DNA, did not answer; EXP, explicit; F, female; HRQoL, health‐related quality of life; IMP, implicit; M, male; Md, median; mHealth, mobile health; Mo, mean; MVPA, moderate‐to‐vigorous physical activity; NRT, nicotine replacement therapy; O, other; PA, physical activity; PFH, Partnership For Health; R, range; RCT, randomized controlled trial; tx, treatment.

^a^ Multi‐domain intervention, repeated in the table according to the domain of intervention.

^b^ Unspecified whether they reported on the sex or the gender of the participants.

### 3.1 Definition of Interventions

We first used the TIDieR checklist to formally describe the intervention programs (Tables 2) and identify gaps in the published descriptions. For several criteria, we had no difficulty finding the relevant information. The rationale of the intervention was generally clear, with explicit reference to theoretical models guiding the intervention design, including the socio‐ecological model [72, 75, 76, 77], self‐determination theory [66, 70, 71, 72, 84], social determination theory [62, 70, 71], theory of reasoned action [73], social cognitive theory [70, 71, 74, 90], theory of planned behavior [80, 81], transtheoretical model [89], principles of motivational interviewing [75, 76, 77, 78], conflict of decision making [79], and change theory [79] Several studies founded their intervention rationale solely on general health guidelines without reference to a specific model of change (e.g., national physical activity recommendations) [83, 86, 87, 88], whereas others failed to articulate any theory‐based rationale for their intervention [82, 85] (Table 2, Column 1).

**TABLE 2:** Intervention description table (based on the TIDieR checklist).

Authors, brief name	Why	What	Who provided	How	Where	When and how much	Tailoring and modifications	How well
		Materials and procedures						Planned and actual
Beight et al. (2024) StepByStep	Addressing PA‐associated outcomes of a distance‐based, multi‐component mHealth and social media behavioral intervention, guided by a Socio‐Ecological Model and Self‐Determination Theory, targeting competence, autonomy, and relatedness	**Materials**: Wearable Fitbit and associated mHealth app. Private, Instagram peer support group. Educational materials on late effects and PA. **Procedures**: Participants wore Fitbits daily, engaged in staff‐moderated Instagram activities and weekly goal setting during Weeks 1–24, then transitioned to self‐directed goal‐setting with reduced staff support and posting frequency in Weeks 25–48.	Coordinating Center Interventionists	Combination of group Fitbit tracking/feedback, weekly individualized goal setting (via text/phone), and peer support/modelling via the private social media group.	Delivered entirely distance‐based/remote	**When**: Total: 48 weeks Intensive: Weeks 1–24 Maintenance: Weeks 25–48 **How much**: Intensive phase: Weekly goal setting; 2–3 staff posts/week; daily Fitbit reminders; badges/awards Maintenance phase: Monthly goal check‐ins; 1 staff post/week; no reminders or badges	**Tailoring**: Individualized goal setting based on the participant's self‐monitored Fitbit step counts. Instagram posts are tailored to participant interests **Modification**: Due to the COVID‐19 pandemic, initial plans for in‐person outcome assessments were modified to be distance‐based	**Planned**: Fidelity assessed by study staff tracking app usage and Instagram engagement. A 25% attrition rate was anticipated. **Actual**: Recruitment goal was achieved. High acceptance of remote evaluation. Final adherence/efficacy data is pending as the study is ongoing.
Berg et al. (2014) No reported name	Addressing modifiable risk factors in young adult survivors of childhood cancer, based on the Theory of Reasoned Action, which posits that behavior is the direct result of intention.	**Materials**: Web‐based module that included tailored graphical depiction and health‐related textual and video messaging **Procedures**: Participants completed a timeline follow‐back reporting (check‐in) every 3 to 4 days regarding minutes exercised, drinks consumed, and cigarettes smoked. Upon check‐in, they were routed to a landing page with tailored graphical depictions and health messages. The deals were offered each time the check‐in was completed.	Self‐delivered	Delivered individually via a Web‐based system. The 12 modules were delivered via email bi‐weekly	Delivered remotely from home [not reported; classified as remote delivery based on description].	**When**: Total: 6 weeks Delivery period: Weeks 1–6 Follow‐up: Week 12 **How much**: 12 modules delivered bi‐weekly Assessments at baseline (Week 0), end of treatment (Week 6), and follow‐up (Week 12)	**Tailoring**: The landing page included tailored graphical depictions of daily exercise, drinking, and smoking. The messages themselves were based on the Theory of Reasoned Action, but were targeted for this population rather than tailored to individuals **Modification**: Not reported	**Planned**: Adherence was assessed by tracking completion of the check‐in assessment over the 12‐module period **Actual**: Adherence averaged 77.8% over the 12 modules
Devine et al. (2020) FitSurvivors	Examining the effects of physical activity, grounded in Social Cognitive Theory, targeting self‐efficacy, goal setting, self‐monitoring, and instruction in proper exercise form	**Materials**: Activity tracker and app, Facebook group **Procedures**: 12 weeks (8 weeks in person, 4 weeks using the app alone) Each session 90 min: 60 min of trainer‐led exercises and 30 min of personalized goal‐setting feedback and education	Qualified trainer	Delivery was a combination of in‐person group sessions and individual mobile app/FitBit use, social media group	Delivered in a gym setting	**When**: Total: 12 weeks In‐person sessions: Weeks 1–8 App and fitness tracker only: Weeks 9–12 **How much**: In‐person sessions: 90 min each: 60 min trainer‐led exercise 30 min personalized goal setting, feedback, and education	**Tailoring**: Tailored intensity according to the participants ratings of perceived exertion or physical limitations **Modification**: Not reported	**Planned**: Staff tracking enrollment, retention, session attendance, app engagement, Facebook group use, and participant satisfaction surveys **Actual**: Limited reach. Suboptimal retention. Session attendance was good and Trainer Satisfaction was very high
Faith et al. (2024) ENHANCE (Empowering New Habits and Nutrition after the Cancer Experience)	Based on the transtheoretical model and health belief model, targeting self‐efficacy and motivation. To mitigate long‐term health risks by addressing poor nutrition and limited PA	**Materials**: Fitbits (for PA tracking), a participant notebook (with educational materials/worksheets), and a to‐scale MyPlate and juice cup picture **Procedures**: 2 sessions combining education, skill‐building, Motivational Interviewing (MI) (Sessions 1–5), and Problem‐Solving Training/Goal Setting (PST) (Sessions 6–12). Specific skills included mindfulness, meal planning, and activity pacing	Four study interventionists (two postdoctoral psychology fellows and two licensed, board‐certified psychologists) who achieved expert‐level MI proficiency prior to delivery	Delivered individually via telehealth (videoconference).	Delivered remotely, in participant's home environment [not reported; classified as remote delivery based on description].	**When**: Total: Up to 16 weeks **How much**: 12 weekly sessions Each session: ~60 min	**Tailoring**: The intervention was individually‐tailored, utilizing motivational interviews and problem‐solving techniques. Content was adapted based on specific nutritional/PA topics raised by participants **Modification**: Not reported	**Planned**: Fidelity assessed using the Session Content‐Fidelity Rating Tool and the Motivational Interviewing Treatment Integrity Coding form **Actual**: 97% of coded MI sessions demonstrated expert proficiency. Clinicians delivered 96.6%–100% of session content
Ha et al. (2025) Making Moves	Guided by the self‐determination theory and three constructs (autonomy, competence, and relatedness)	**Materials**: 8 self‐directed educational modules online (including educational content in short text and videos and exercise tips) and a Fitbits (for PA tracking) **Procedures**: 8 weeks of online physical activity program, self‐paced and self‐directed.5 telehealth exercise consultations with an Accredited Exercise Physiologist (AEP) (at T0, T2, T3, T4 and optionally at T1)	Videos were created and filmed by exercise physiologists in training and approved by an Accredited Exercise Physiologist. Personal telehealth exercise consultations were conducted with and AEP.	Online program, self‐paced and consultation via telehealth (videoconference).	Delivered remotely, in participant's home environment	**When**: Total: 8 weeks **How much**: 8 self‐paced videos and 4 or 5 consultations (length of consultations unspecified)	**Tailoring**: The intervention was individually‐tailored based on participants needs, goals and barriers. To ensure the program age‐appropriateness, content, readability and exercise have been tailored slightly to address three age groups. **Modification**: *NA* [research protocol]	**Planned**: *Implementation data, feasibility, recruitment rate, and retention rate*
Keats et al. (2008, 2009) No reported name	Guided by the Theory of Planned Behavior, targeting attitude, subjective norm, perceived behavioral control, and intentions	**Materials**: Educational materials, PA protocols (aerobic, strength, flexibility), RPE scale. **Procedures**: The first 8 weeks consisted of a 30‐min educational session, 45 min of aerobic training, and 15 min of core strength and flexibility training. Participants were closely monitored to ensure that they were working within safe boundaries. The final 8 weeks included a variety of noncompetitive physical activities. No formal lifestyle education was provided during these sessions.	Trained instructor or trained fitness professional	Delivery was via structured weekly, In‐person, group settings	Conducted in a group setting at the University of Calgary's Faculty of Kinesiology	**When**: Total: 16 weeks **How much**: Weekly sessions. Weeks 1–8: 30 min education + 60 min structured exercise Weeks 9–16: 90 min non‐competitive physical activity (no formal education)	**Tailoring**: Participants were instructed to participate at an intensity they felt most comfortable using the RPE scale. Fitness data was kept confidential and used for objective, personalized feedback and self‐monitoring guide. **Modification**: No major unplanned deviations were reported.	**Planned**: Feasibility assessed by measuring recruitment, adherence (attendance), and retention. **Actual**: High adherence: Average attendance, High retention
Le et al. (2017) No reported name	Addressing low exercise rates among childhood cancer survivors who face a high risk of late complications	**Materials**: A Fitbit One activity tracker. Access to the interactive activity tacker website and the activity tracker brochure **Procedures**: Participants were instructed to wear the Fitbit One daily for 6 months clipped to their waist. They were taught how to register the Fitbit, log activity online, and upload their data. They were educated about MVPA and instructed to adhere to Centers for Disease Control and Prevention exercise recommendations	Self‐delivered	Delivery was individual, primarily home‐based exercise	Home‐based	**When**: Total: 6 months **How much**: Device worn daily	**Tailoring**: Not reported **Modification**: Not reported	**Planned**: Fidelity/Adherence was assessed by tracking the number of days the Fitbit was worn for at least 15 min using the interactive website **Actual**: *Retention rate was 79%. Participants wore the Fitbit an average of 19.0 days/month (months 1–3) and 15.0 days/month (months 4–6)*
Long et al. (2018) No reported name	Addressing the feasibility and effectiveness of regular exercise in minimizing or reversing treatment‐induced deficits that predispose AYA survivors, based on health guidelines for recommended daily PA	**Materials**: Combination of free weights, machine weights, body weight materials, and ergometers. Participants were provided with an accelerometer and a journal to record daily progress **Procedures**: 90‐min sessions consisting of circuit‐based resistance work and moderate intensity aerobic interval training using different ergometers	Exercise physiologist	Delivered primarily face‐to‐face training sessions	Held at a number of venues chosen for their proximity to the participant's home addresses to aid adherence	**When**: Total: 24 weeks **How much**: Sessions: 90 min each Schedule: 3 sessions per week Minimum attendance: 2 sessions per week required for inclusion	**Tailoring**: Tailored for each individual based on their baseline strength, endurance, and aerobic capacity testing results **Modification**: Not reported	**Planned**: Fidelity was monitored by the supervising exercise physiologist marking off exercises on an individual training log **Actual**:
Mendoza et al. (2017) No reported name	The intervention was guided by Self‐Determination Theory, aiming to increase competence, autonomy, and relatedness	**Materials**: A wearable PA‐tracking device and its mobile phone‐based app. A private, invitation‐only Facebook group **Procedures**: Participants wore the Fitbit Flex and were encouraged to use the Facebook group as a peer‐based virtual support group. Research staff moderated the Facebook group, provided badges, and sent affective text messages every other day. Staff contacted participants weekly to set and gradually increase step count goals	Research staff	Delivered individually (Fitbit/app self‐monitoring, tailored goals) and in a group (Facebook forum). Delivery modes included the wearable device, mHealth app, and distance communication (telephone/text message)	Delivered primarily via mHealth/online platforms (Fitbit app, Facebook), allowing participants to engage from their home environment.	**When**: Total: 10 weeks **How much**: Weekly goal‐setting contact from research staff (from Week 2) Affective text messages every other day	**Tailoring**: The intervention was personalized by having participants set individualized daily step goals **Modification**: Not reported	**Planned**: Fidelity was monitored through activity tracker wear time **Actual**: Activity tracker worn for 71.5% of days, meeting the “majority of days” criterion^a^
Miller et al. (2021) GECCOS (Green Exercise for Childhood Cancer Survivors) pilot study	Promoting outdoor vs. indoor exercise intensity and long‐term motivation and behavior in AYACS, based on the Self‐Determination Theory. The design of the exercise sessions was informed from a community partner and three focus groups	**Materials**: Actigraph GT3x accelerometers. Instructor Manual provided with a specific and detailed description of the theory‐based program. Sessions outlines are provided and provide a structure for each session and specific ways that the session should support autonomy, competence and relatedness. **Procedures**: Four group exercise sessions (two indoor, two outdoor) focused on walking. Each session included an introduction, 30–50 min of exercise, promoted socializing among participants, and a meal provided afterward	Delivered by two young adult cancer survivors hired as peer leaders and were supported by guides from the community partner and the research team.	Delivered in group exercise sessions, primarily face‐to‐face. Participants were allowed to bring a friend or family member.	Outdoor sessions: A large park in Minneapolis (in community partnership with the Loppet Foundation). Indoor sessions: Tunnels and skyways at the University of Minnesota	**When**: Total: 4 sessions delivered over 2 separate weeks Schedule: Sessions delivered 1 month apart **How much**: Sessions: 2 indoor and 2 outdoor Session length: 30–50 min each	**Tailoring**: Due to the group nature of the exercise session, tailoring to individual preferences was not possible Exercise modifications are provided in the manual—for example: **Modification**: One outdoor session for one group had to be re‐scheduled due to thunderstorms	**Planned**: Attendance was taken at each session **Actual**: 19 survivors were consented, meeting recruiting goals. Attendance varied: five participants attended all four sessions
Rueegg et al. (2017, 2023), Jung et al. (2023) SURfit	The program aimed for sustained behavior change toward an active lifestyle based on the international recommendations of healthy physical activities from the Centre of Disease Control and Prevention	**Materials**: Pedometers, a self‐administered web‐based daily activity diary with immediate graphical feedback. The web platform was hosted by REDCap. **Procedures**: Intervention participants were asked to add 2.5 h of intense PA/week to their baseline activity and were advised to reduce screen time by 25%. Sessions included a standardized assessment of preferences/barriers and a motivational interview to define individualized PA activities.	Counseling provided by physiotherapists. They were trained prior to the study start by a sports physician. Counseling/coaching was also provided by a “personal coach” (physiotherapist) via phone contacts	Delivered individually and was self‐implemented into the survivor's daily living	Home‐based and clinic	**When**: Total: 12 months **How much**: Face‐to‐face contacts: 4 (baseline, 3, 6, and 12 months) Phone contacts: 6 (months 1, 2, 4, 5, 8, and 10) Reminders: Email/phone prompts after ≥ 3 days without diary entries	**Tailoring**: Individualized PA programs were developed together with a physiotherapist. Activities were defined based on the survivor's preferences, motivation, barriers, and initial exercise test results. Goals (steps, exercise type) were adapted to the individual. **Modification**: No major unplanned deviations were reported.	**Planned**: Fidelity/Compliance was monitored by physiotherapists during follow‐up contacts
Salchow et al. (2021) Motivate AYA (MAYA) trial	The trial aimed to show the effect of counseling on improving vigorous physical activity behavior to reduce the high risk of cardiovascular events, based on the Transtheoretical Model (TTM).	**Materials**: General physical activity guidelines for cancer survivors were provided to the control group. The intervention utilized the International Physical Activity Questionnaire (IPAQ) to measure activity and tailored information based on individual needs. Counseling content was based on an adaptation of the TTM **Procedures**: The intervention consisted of a semi‐structured interview (counseling) and telephone follow‐up. Procedures included: documenting physiological limitations, assessing PA behavior before and after cancer treatment, discussing the benefits of vigorous physical activity, referring to current PA guidelines, and developing a specific, tailored personal exercise plan	Sports scientist	Delivered individually, and telephone counseling	At home [not reported; classified as remote delivery based on description].	**When**: Total: 12 weeks Follow‐up assessment: 52 weeks **How much**: Initial semi‐structured interview: 60 min (Week 0) Telephone counseling sessions: Weeks 1, 3, and 12	**Tailoring**: The intervention was personalized based on the individual needs of the participant and stage of readiness to tailor the content and goals **Modification**: Not reported	**Planned**: Fidelity/Adherence was assessed by the sports scientist **Actual**:
Williamson‐Lewis et al. (2023) No reported name	To characterize the feasibility and identify patterns of use of a wearable activity tracker among adolescent cancer survivors, based on the Social Cognitive Theory suggesting that health behavior change occurs in the context of self‐management skills.	**Materials**: A Fitbit Flex device. Access to the Fitbit.com account and the Fitbit application on the participant's mobile device. Participants and parents also received an educational handout adapted from the American Heart Association outlining PA recommendations and health benefits **Procedures**: Participants were instructed to wear the Fitbit daily. At enrollment, staff assisted in setting up the Fitbit.com account and installing the app. Participants selected a goal of 10,000 steps per day. Researchers downloaded synced daily summary data (steps and active minutes) through Fitbit's API for 1 year. No additional contacts were made to encourage use after setup	Self‐administered	Delivered individually.	At home or in spaces conducive to physical activity [not reported; classified as remote delivery based on description]	**When**: Total tracking period: 12 months Initial assessment period: First 12 weeks Follow‐up survey: Mean 12.5 months **How much**: Daily activity tracking	**Tailoring**: Participants selected their own goal of 10,000 steps per day **Modification**: Not reported	Not reported
Quidde et al. (2016) INAYA (Improved Nutrition in AYAs) trial	The intervention aimed to modify lifestyle risk factors	**Materials**: The 3‐day dietary record used to collect baseline and follow‐up data. The intervention content was based on the recommendations of the German Nutrition Society guidelines. **Procedures**: AYAs filling out the 3‐day dietary record; assessment of nutritional behavior; providing individually tailored recommendations based on the German Nutrition Society guidelines.	Registered dietitian	Delivered by face‐to‐face at baseline and after 12 weeks, as well as by telephone for Weeks 2 and 6 (individual sessions)	Counseling occurred at the Hubertus Wald Tumour Center, University Cancer Center Hamburg.	**When**: Total: 12 weeks **How much**: Total contacts: 4 Face‐to‐face counseling: 60 min (Weeks 0 and 12) Telephone counseling: 30 min (Weeks 2 and 6)	**Tailoring**: Recommendations were modified and individually tailored to the needs of every AYA, based on results and reported nutrition problems **Modification**: Not reported	**Planned**: Adherence/Compliance was monitored by tracking attendance **Actual**: 73.9% at Week 6, and 69.6% at Week 12. The overall attrition rate was 30%
Hollen et al. (2013) Decision KEYS for Balancing Choices	To test a cognitive‐behavioral skills program to enhance decision‐making skills aimed at difficult decisions related to substance use risk behaviors, based on the Janis and Mann theory of decision‐making	**Materials**: Five CD‐ROM modules. A hard copy of the workbook was mailed. The Risk Behavior Facts Sheet was used for tailored counseling. The Usual Care group received a neutral sham CD‐ROM on study skills. **Procedures**: (1) Initial one‐on‐one counseling by the nurse practitioner using the tailored Risk Behaviors Facts Sheet. (2) Viewing CD‐ROM modules at baseline. (3) Receiving e‐boosters (CD‐ROM components) at 2, 4, and 6 months. (4) A 9‐month telephone booster, expanded for high riskers, repeating the tailored counseling	Clinic nurse practitioners	Delivered individually. Modes included face‐to‐face counseling/interview, CD‐ROMs, telephone contact	Data collection occurred during clinic visits (baseline/12 months) and off site from the teen's home/library computer (6 months)	**When**: Total: 12 months **How much**: Initial visit/counseling: Week 0 e‐boosters: Months 2, 4, and 6 Telephone booster: Month 9 Total: 7.5 contact hours	**Tailoring**: The intervention was tailored. Counseling used a “tailored” Substance Use Risk Behaviors Facts Sheet related to the individual's late effects of cancer/cancer treatment. Content was tailored by risk behavior status **Modification**: Not reported	**Planned**: Fidelity/Adherence was assessed using two Protocol/Study Checklists completed by the nurse practitioner/study coordinator
Asfar et al. (2010), Klesges et al. (2015) St. Jude Cancer Survivors Tobacco Quit	To examine the long‐term efficacy of an intensive Quit Line cessation intervention, was based on Social Cognitive Theory and Self‐Determination Theory	**Materials**: Nicotine Replacement Therapy, primarily patches and gum. A “Curbing your Cravings” brochure providing detailed instructions regarding proper use of NRT. Supplemental rate reduction materials for participants who failed to quit. **Procedures**: Six weekly, 15–30 min individualized telephone counseling sessions. The sessions included three phases: (1) preparing to quit (cue breaking, rate reduction), (2) the quitting process (setting a quit date, NRT use), and (3) relapse prevention (identifying high‐risk situations). A specific rate reduction protocol was implemented for those unwilling to reset a quit date.	Trained experienced counselors in tobacco dependence, underwent intensive full‐time three‐week training covering motivational interviewing, behavioral theories, pharmacotherapies, and counseling protocol.	Delivered individually via telephone‐based Quit Lines	At home or space with phone access [not reported; classified as remote delivery based on description].	**When**: Total: 8 weeks **How much**: Telephone counseling: Up to 6 sessions **Proactive group**: 8 weeks of NRT supply (two 4‐week mailings) **PI/Reactive group**: 2‐week NRT starter pack; encouraged to obtain remaining 6 weeks	**Tailoring**: The NRT patch dose was tailored to the participant's smoking rate. The behavioral intervention was tailored to CCSs by identifying their high‐risk status and linking smoking to potential exacerbation of late effects **Modification**: Not reported	Not reported
Emmons et al. (2005, 2009), Park et al. (2006) Partnership for Health (PFH) Study	To minimize preventable risk factors (smoking) in the high‐risk population of childhood cancer survivors, guided by the Social Ecological Model and integrated principles from various theories of behavior change (including motivational interviews)	**Materials**: PC Group: Tailored and targeted written materials. Free nicotine replacement therapy Self‐help Group: A letter about smoking risks from study physicians and the “Clearing the Air: How to Quit Smoking and Quit for Keeps” cessation manual **Procedures**: PC Group: Up to six individualized telephone calls were provided within a 7‐month period. Counseling used Motivational Interviewing principles, emphasizing smoker's choice and self‐efficacy. Call content was tailored to the participant's stage of readiness to quit smoking, nicotine replacement therapy was provided upon readiness to make a serious quit attempt. Self‐help Group: Participants received written materials by postal service mail.	The intervention was delivered by a peer counselor trained childhood cancer survivor. Counselors received week‐long training	Delivered individually via proactive telephone counseling.	At home or space with phone access [not reported; classified as remote delivery based on description].	**When**: Total: 7 months **How much**: Up to six calls with peer counselor	**Tailoring**: Not reported **Modification**: Not reported	**Planned**: Fidelity was monitored by having a supervisor review 10% of each counselor's calls (which were taped) to ascertain treatment fidelity and appropriate use of motivational interviewing techniques

^a^ Entries modified following reviews of entry using Notebook LM.

The mode of intervention delivery was well documented and described. Four interventions included both group and individual components [66, 72, 74, 83], whereas 11 interventions were delivered individually [62, 70, 71, 73, 75, 76, 77, 78, 79, 82, 85, 86, 87, 88, 89, 90], and two were delivered in group settings [80, 81, 84]. Three interventions utilized both in‐person and remote delivery [74, 79, 85], three were delivered in person [80, 81, 83, 84], and 11 were delivered remotely, using social media [66, 72, 74], videoconference or phone [62, 70, 71, 72, 75, 76, 77, 78, 85, 86, 87, 88, 89], web‐pages or apps [62, 66, 73, 74, 82, 90], message texts or emails [66, 72, 73, 79], or CD‐ROM [79] (Table 2, column 4). Intervention intensity and durations were reported in almost all retrieved items, except in four cases where information on the required weekly time commitment was missing [62, 66, 72, 73]. In one intervention, intensity and duration were tailored to participants, but information on the range for either parameter was lacking [66].

Intervention tailoring was addressed in all but two interventions [73, 82]. Most often, communication was tailored according to individual motivation stage (such as stage of readiness to change [75, 76, 77, 89], or motivation level [86, 87, 88]) or to individual behavior profile (such as risky behavior status [70, 71, 79]). Other programs tailored their content according to physical limitations [74, 84], current habits and levels [62, 66, 83, 85], preferences [86, 87, 88] and values [78], perceived physical exertion [74, 80, 81], individualized goals [66, 72], or age [62] (Table 2, Column 7).

Descriptions of intervention procedures were generally well documented in a way that would allow replication. Procedures often included clearly defined phases and contact schedules, with interventions detailing baseline assessments, intervention timeline, and follow‐up assessments. Overall, most interventions reported enough procedural, activity, and process information to permit implementation by other teams (Table 2, Column 2).

However, descriptions of intervention materials were often incomplete, with several studies omitting essential details needed for replication. While many reported the broad categories of resources provided, such as nicotine replacement therapy [70, 71, 75, 76, 77], wearable activity trackers [62, 66, 72, 74, 78, 82, 83, 84, 86, 87, 88, 90], web‐based or app‐based self‐monitoring platforms [74, 82, 83, 86, 87, 88], printed or digital educational resources [62, 70, 71, 72, 73, 79], and access to private social media groups [66, 72, 74], the specifics about content, format, and delivery were frequently missing. In some cases, materials were mentioned, including participant notebooks with worksheets and homework assignments [78, 83], personalized health profiles [75, 76, 77] or exercise plan [89], but these were not reproduced or linked for readers. Only one program [84] provided a training manual for providers, outlining the structure, content, location, and materials for each session in their Supporting Information. Educational packets adapted from guidelines [72, 78, 82, 85, 90], and training modules [62, 73, 79], were referenced without being made available. Where social media posting or individual text messages were described, one study provided examples [72], however message scripts, instructional curriculum, content libraries, or social media moderation protocols were lacking in all items using social media posting [66, 72, 74] (Table 2, Column 2).

As for location, the information provided was not systematically explicitly reported and was classified based on description in 6 (35.3%) of the 17 programs [71, 73, 76, 78, 89, 90]. For remote or home‐based interventions [62, 66, 70, 71, 72, 73, 75, 76, 77, 78, 79, 82, 85, 86, 87, 88, 89, 90], few details were provided regarding when and where participants should practice (e.g., at home, in a quiet private room, or in a space suitable for exercise). Two intervention locations were vaguely described as a “gym setting” [74] or “venues” [83]. In contrast, two other intervention sites were clearly specified, including both the type of space and the physical location (building and city) [80, 81, 84] (Table 2, Column 5). As for the name of the intervention, 35.3% of the intervention programs (*n* = 6 out of 17) did not mention a name for their program, and only provided a short description [66, 73, 80, 81, 82, 83, 90] (Table 2, Column 1). Some information about intervention providers and their training was available in all but two of the 17 interventions. Twenty‐nine percent (*n* = 5 out of 17) of programs [70, 71, 75, 76, 77, 78, 84, 86, 87, 88] provided thorough information about the providers, including their professional background and the specific training provided for the purpose of the intervention. However, in 41% (*n* = 7 out of 17) of interventions [66, 74, 79, 80, 81, 83, 85, 89], the details provided were very limited, lacking in specificity about the background or training of the providers (such as a “qualified trainer” [74], or “sport scientist” [89]). Three interventions were self‐delivered [73, 82, 90] (Table 2, Column 3). Only three programs of the 17 programs (17.6%) reported modifications in line with the TIDieR framework: one due to COVID‐19‐related adjustments [72], one involving a change from outdoor to indoor sessions because of weather conditions [84], and one explicitly stating that no modifications were made [86, 87, 88] (Table 2, Column 7). Finally, treatment fidelity, both planned and observed, was largely overlooked. Only four out of 17 (23.5%) interventions [77, 78, 79, 83] explicitly mentioned planned monitoring strategies, such as call recording [77], supervision meeting [77], content fidelity tool [78], integrity coding form [78] or the use of supervision checklists [79, 83]. Only one of these four items reported on the actual percentage of fidelity of the intervention delivered [78]. Interestingly, 65% (*n* = 11 out of 17) reported only on the planned and actual attendance, adherence, retention or engagement to the intervention as a marker of intervention fidelity [62, 66, 72, 73, 74, 80, 81, 82, 84, 85, 86, 87, 88, 89]. Two reports did not even address the planned nor the actual attendance, adherence, retention or engagement of the intervention delivery [70, 71, 90] (Table 2, Column 8).

### 3.2 Behavior Change Techniques

Behavior change techniques are documented in Table 3, with each intervention function and sources of behavior change for each item, using the BCW framework. As documented in Figures 3 and 4, on average, programmes incorporated about four of the nine recognized functions and engaged approximately four out of the six COM‐B sources of behavior change. Three interventions mobilized only two of the three COM‐B pillars; one excluded capacity [82], while two excluded opportunity [78, 85]. All the other interventions engaged at least one aspect of the three pillars, with one using all two aspects of each pillar [74].

**TABLE 3:** Intervention functions and COM‐B behavioral sources identified in included studies.

Authors	Behavior change intervention functions and descriptions	Source of behavior change and descriptions
Physical activity		
Beight et al. (2024)	**Education**: Provided educational materials on late effects of childhood cancer and importance of PA **Persuasion**: Interventionists posted encouraging advice and motivational discussion questions **Incentivization**: Awarded weekly announcement of badges/awards **Training**: **conducted i**ndividualized goal setting sessions teach skills for setting personalized steps **Environmental restructuring**: Used a private Instagram group to structure the social environment **Modelling**: Encouraged peer sharing of PA content (photos/videos) within the group **Enablement**: Provides activity tracker and mHealth app for tracking and feedback	**Psychological capacity**: Enhanced competence and self‐monitoring skills via app tracking, knowledge and skills, and goal setting **Physical opportunity**: Provided activity tracker **Social opportunity**: Private social media peer group provided relatedness and community support **Reflective motivation**: Targeted intrinsic motivation by fostering autonomy and competence beliefs with goal setting and habit tracking **Automatic motivation**: Badges and awards provide positive, external reinforcement
Berg et al. (2014)	**Education**: Provided textual and video messaging and module content **Incentivization**: Offered deals for healthy goods and services each time they completed the bi‐weekly health behavior check‐in **Enablement**: Provided deals also increase the means (financial opportunity) for accessing healthy activities	**Psychological capacity**: Focused on improving coping skills necessary to maintain health behaviors **Physical opportunity**: Offered deals for healthy goods and services (e.g., health food discounts, wall climbing entries, massages) in their local area, physically increasing access to healthy options. **Reflective motivation**: Required timeline follow‐back reporting of activity, drinking, and smoking for self‐monitoring and tracking **Automatic motivation**: Offered deals each time they completed the health behavior check‐in, creating expectation of reward
Devine et al. (2020)	**Education**: Educational session, focusing on behavior change strategies, information about exercise and nutrition, and healthy habits and lifestyle **Persuasion**: Communicated through peer support group to induce positive reinforcement and motivation **Incentivization**: Achievements unlocked when meeting a goal, shared with the group to highlight participants success and progress **Training**: Physical training session to teach participants to gradually increase resistance, repetitions and minutes of MVPA overtime **Environmental restructuring**: Tips shared with participants on how to change their social and physical environment to promote sustainable change **Modelling**: Peer modelling and support through a private Facebook group for participants **Enablement**: Provision of an activity tracking device and tracker app	**Physical capacity**: Trainer‐led exercise and instruction on proper form **Psychological capacity**: Increased knowledge and cognitive skills through information on health knowledge, goal setting, and self‐monitoring skills **Physical opportunity**: Provision of activity tracker, mobile app, and a supervised training location **Social opportunity**: Group sessions and app social feed **Reflective motivation**: Guided by social cognitive theory, explicitly targeting self‐efficacy, self‐monitoring, goal setting, evaluation and reflection **Automatic motivation**: App awarded achievements and points for meeting activity goals
Faith et al. (2024)	**Education**: Informational session reviewing daily aerobic and strength building guidelines and definitions of MVPA **Training**: Sessions included skill‐building, addressing time management and habit building. Skills training also included gradual shaping. **Enablement**: Participants were provided with an activity tracker, which functioned as an accessible measurement tool.	**Psychological capacity**: Aimed to improve self‐efficacy related to PA and health behaviors via skill‐building and goal setting **Physical opportunity**: Activity trackers were provided, making objective monitoring of physical behavior readily available in the participant's environment **Reflective motivation**: **F**ocused on values‐based healthy lifestyle decision‐making, activity planning, and identify individualized goals
Ha et al. (2025)	**Education**: Educational modules provided content on different subjects, including active living, best practice of training. **Training**: Online modules included teaching physical skills, addressing progression, strength, endurance, flexibility, balance, and coordination **Enablement**: Provision of an activity tracker	**Physical capacity**: Improvement of physical fitness through aerobic, strength, and flexibility training **Psychological capacity**: Imparting cognitive abilities in recognition of barriers, instruction in self‐monitoring, and program planning skills **Physical opportunity**: The provision of an activity tracker **Social opportunity**: Encouraged family and friends to follow the videos to learn new skills together **Reflective motivation**: Targeted self‐management skills such as goal setting, and monitoring
Keats et al. (2008, 2009)	**Education**: Provides information on short and long‐term benefits of PA and active living **Training**: Teaches and rehearses self‐management skills such as goal‐setting and self‐monitoring. Aerobic training, and 15 min of core strength and flexibility training. **Environmental restructuring**: Provides a noncompetitive, supervised, and fun environment in a university setting **Modelling**: Survivor mentors and the peer group were enlisted to provide support and positive examples **Enablement**: Provided physical spaces for engaging in physical activity	**Physical capacity**: Improvement of physical fitness through aerobic, strength, and flexibility training **Psychological capacity**: Imparting cognitive abilities in recognition of barriers, instruction in self‐monitoring, and program planning skills **Physical opportunity**: Provision of a safe, specialized location for group exercise **Social opportunity**: Fostering group cohesion and social support among survivors, mentors, and peers **Reflective motivation**: Targeted self‐management skills (goal setting, program planning, self‐monitoring, perceived behavioral control)
Le et al. (2017)	**Incentivization**: Provided gift cards monthly for achieving minimum daily wear‐time compliance **Enablement**: Provided a motivational activity tracker and access to its interactive website for monitoring progress.	**Physical opportunity**: The provision of an activity tracker may provide the means to perform a behavior **Reflective motivation**: Activity tracking through the activity tracker features **Automatic motivation**: the incentives participants receive for completing the baseline and follow‐up evaluations, as well as for wearing the Fitbit, may drive behavior
Long et al. (2018)	**Training**: Structured exercise sessions covering resistance and aerobic training. **Enablement**: Provided an activity tracker	**Physical capacity**: Direct enhancement of muscular strength and submaximal aerobic capacity via prescribed exercise protocols. **Physical opportunity**: Different venues to perform activity and provision of Fitbit **Reflective motivation**: Habit tracking (activity tracker)
Mendoza et al. (2017)	**Persuasion**: Encouragement provided by sending affective text messages every other day to encourage and remind participants about their PA goals **Incentivization**: Provision of badges (awards) every week for PA and participation achievements **Training**: Participants set daily step goals and gradually increase those goals, which guided goal setting imparts planning skills necessary for behavior change. **Environmental restructuring**: Established a private, invitation‐only Facebook group, which functioned as a peer‐based virtual support group **Modelling**: Showcasing successful PA engagement and providing peer examples for others to emulate **Enablement**: Participants were provided with an activity tracker wearable wristband and mHealth app to track PA metrics and progress toward personalized goals	**Psychological capacity**: Providing weekly assistance to help set and gradually increase daily step goals, imparting skills and knowledge for planning **Physical opportunity**: An activity tracker and mHealth app were provided to all participants **Social opportunity**: A private Facebook group was established, serving as a peer‐based virtual support group to provide encouragement and positive reinforcement **Reflective motivation**: The Fitbit and mHealth app allowed participants to set personalized step count goals, supporting conscious planning and autonomy **Automatic motivation**: Weekly badges (awards) were provided via Facebook for achievement to motivate participants through competition and anticipated reward
Miller et al. (2021)	**Training**: Structured exercise sessions, providing participants with practice and skill development in maintaining exercise duration and intensity **Environmental restructuring**: Provision on an accelerometer to track their weekly MVPA. **Modelling**: Two young adult survivors were hired as peer leaders to pace the sessions and interact with participants, demonstrating the desired exercise behavior and leadership (peer leader) **Enablement**: Provision of a list of locations for inexpensive spin classes around them, reducing access barriers. Provision of training material to participants.	**Physical capacity**: Structured exercise, providing participants with practice and skill development in maintaining exercise duration and intensity **Physical opportunity**: Varied physical settings by comparing Outdoor exercise with Indoor exercise sessions, and provided with an activity tracker **Social opportunity**: Group exercise sessions were established where peer leaders and research guides promoted socializing, and participants were allowed to bring a friend or family member **Reflective motivation**: Self‐reflection and questions about the training session involving conscious evaluations and plans
Rueegg et al. (2017, 2023), Jung et al. (2023)	**Environmental restructuring**: Participants were advised to reduce inactive behavior, such as television viewing and computer games, with the aim of reducing media‐related sedentary time **Enablement**: Participants were provided with an activity tracker and a web‐based daily activity diary to track steps and exercise, reducing barriers to self‐monitoring and acting as motivational tools	**Psychological capacity**: Enhancing Psychological Capability By Improving Decision‐Making And Mental Readiness For Behavior Change Through Motivational Interviews **Physical opportunity**: Participants Were Provided With An Activity Tracker To Encourage And Track Daily Steps. **Reflective motivation**: Participants Were Advised On Reducing Screen Time And Used The Web‐Based Daily Activity DIARY For Self‐Monitoring And Reflection On Strenuous Exercise, Step Counts, Media Time, And Mood
Salchow et al. (2021)	**Education**: Informed about the benefits of regular physical activity, discussed the participant's personal health situation, and referred them to the current physical activity guidelines for cancer survivors **Persuasion**: The intervention involved a motivational talk during the semi‐structured interview, which included recalling the positive impacts of former PA behavior and imagining future PA and its possible positive impacts **Environmental restructuring**: Focused on managing the context of sedentary behavior by providing advice on reducing screen time and developing a strategy to avoid relapse to inactive behavior **Enablement**: Local possibilities for PA and exercises were assessed and provided to participants	**Psychological capacity**: Provided with knowledge and skills about the benefits of regular activity **Physical opportunity**: Assessed local possibilities for PA and exercises, including oncological training‐ and exercise therapy, to match the personalized plan to the participant's environment. **Reflective motivation**: Involved developing a specific, tailored personal exercise planning, and goal setting
Williamson Lewis et al. (2023)	**Education**: Participants received an educational handout outlining PA recommendations and guidelines, and associated health benefits **Enablement**: Participants received an activity tracker and were also offered a 6‐month family membership to a local gym to ensure access to an exercise facility	**Psychological capacity**: Educational material to increase their knowledge and skills **Physical opportunity**: Provision of an activity tracker and gym membership to reduce barriers of the physical environment. **Reflective motivation**: Demonstration of engagement in conscious evaluation through goal setting and activity tracking
Nutrition		
Faith et al. (2024)	**Education**: Informational session reviewing dietary guidelines, food groups, and portions **Training**: Sessions addressed mindful eating, appetite awareness/monitoring satiety, and meal planning and preparation **Environmental restructuring**: Addressed dining with friends, coworkers, classmates, and dining on‐the‐go and/or in restaurants, facilitating planning for social food contexts, thus enabling changing social context	**Psychological capacity**: Intervention components included skills for satiety training, stimulus control, and meal planning and preparation. **Reflective motivation**: Monitoring long‐term goals and trends, goal setting, habit building, time management, schedule **Automatic motivation**: focused on avoiding eating in the absence of hunger, and value‐driven vs. emotion‐driven healthy choices, targeting impulsive eating.
Quidde et al. (2016)	**Education**: Counseling based on recommendations to improve nutritional status and behavior	**Psychological capacity**: The core intervention provided the knowledge of German Nutrition Society recommendations **Reflective motivation**: required participants to engage in conscious planning based on individually tailored recommendations
Alcohol consumption		
Berg et al. (2014)	**Education**: The program provided textual and video messaging and module content **Incentivization**: Participants were offered deals for healthy goods and services each time they completed the bi‐weekly health behavior check‐in. This structure was modeled after commercial “daily deal” programs **Enablement**: The provided deals also increase the means (financial opportunity) for accessing healthy activities.	**Psychological capacity**: Focus on improving coping skills necessary to maintain health behaviors **Physical opportunity**: Offered deals for healthy goods and services (e.g., health food discounts, wall climbing entries, massages) in their local area, physically increasing access to healthy options. **Reflective motivation**: Implicitly targeted, their level of confidence in and importance of health behaviors, required timeline follow‐back reporting of activity, drinking, and smoking for self‐monitoring and habit tracking **Automatic motivation**: Incentive structure modeled where participants were offered deals each time they completed the health behavior check‐in, creating expectation of reward
Hollen et al. (2013)	**Education**: The decision aid provided information about cancer/cancer treatment late effects **Persuasion**: Use of cancer survivor testimonial to induce feelings and stimulate action in participants **Training**: Cognitive‐behavioral skills program focused on decision making, teaching theory related to quality decision making. Participants also received assistance for implementing the exercise plan **Modelling**: Use of live action videos to portray positive role modelling	**Psychological capacity**: Cognitive‐behavioral skills program focused on decision making and provided balance sheet exercises in a workbook for practice **Social opportunity**: Use live action videos with diverse positive role models to illustrate decision‐making and role‐play refusal strategies **Reflective motivation**: Aimed to increase quality decision making through conscious planning and adherence to seven decision‐making criteria
Tobacco consumption		
Berg et al. (2014)	**Education**: The program provided textual and video messaging and module content on the risk of smoking **Incentivization**: Participants were offered deals for healthy goods and services each time they completed the bi‐weekly health behavior check‐in. This structure was modeled after commercial “daily deal” programs **Enablement**: The provided deals also increase the means (financial opportunity) for accessing healthy activities.	**Psychological capacity**: Focus on improving coping skills necessary to maintain health behaviors **Physical opportunity**: Offered deals for healthy goods and services (e.g., health food discounts, wall climbing entries, massages) in their local area, physically increasing access to healthy options. **Reflective motivation**: Implicitly targeted, their level of confidence in and importance of health behaviors, required timeline follow‐back reporting of activity, drinking, and smoking for self‐monitoring and tracking **Automatic motivation**: Incentive structure modeled where participants were offered deals each time they completed the health behavior check‐in, creating expectation of reward
Asfar et al. (2010), Klesges et al. (2015)	**Education**: Use of brochures to increase knowledge and understanding about smoking cessation and withdrawal effects **Training**: Developing effective self‐regulatory skills through strategies such as self‐monitoring, goal setting, skills training, modeling, and self‐reinforcement **Environmental restructuring**: Implementing smoking rate reduction strategies, breaking the cues related to smoking, engaging in a stop smoking “ritual” prior to quitting, and enhancing social support **Modelling**: Explicitly stated modeling as a strategy by authors for developing effective self‐regulatory skills necessary for cessation [detailed example not provided in the intervention description] **Enablement**: Provision of adjunctive nicotine replacement therapy to participants	**Psychological capacity**: Development effective self‐regulatory skills for cessation through strategies such as self‐monitoring, goal setting, and skills training **Physical opportunity**: Provision of nicotine patches, and information about nicotine replacement therapy, making medication physically accessible **Social opportunity**: The preparing to quit phase includes enhancing social support [detailed example not provided in the intervention description] **Reflective motivation**: Focuses on developing effective self‐regulatory skills through strategies such as self‐monitoring, goal setting, skills training, modeling, and self‐reinforcement **Automatic motivation**: Provided tool to breaking the cues related to smoking, engaging in a stop smoking “ritual” prior to quitting
Emmons et al. (2005, 2009), Park et al. (2006)	**Education**: Increased knowledge about health risk of smoking with targeted intervention materials, including the cost and benefit of quitting **Training**: Focused on behavioral cessation strategies such as setting a quit date and coping with nicotine withdrawal based on readiness to change stages **Environmental restructuring**: Free nicotine replacement therapy was provided to participants (and their partners/spouses) who were ready to make a serious quit attempt, increasing social support to quit **Modelling**: Counseling was peer‐delivered by trained individuals who were themselves childhood cancer survivors, serving as role models **Enablement**: Free nicotine replacement therapy was provided to participants (and their partners/spouses) who were ready to make a serious quit attempt, reducing barriers and means	**Psychological capacity**: Focused on developing behavioral cessation strategies, such as setting a quit date, preventing relapse and coping with nicotine withdrawal. **Physical opportunity**: Free NRT was made available to PC participants who were ready to make a serious quit attempt, physically reducing a barrier to using medication that aids quitting **Social opportunity**: Counseling with trained childhood cancer survivor providing sustained social support and establishing a supportive social context for change, and NRT provision to spouse, enhanced personal social support and context **Reflective motivation**: Goal set to **t**ailor to the participants' stage of readiness to quit smoking
Hollen et al. (2013)	**Education**: The decision aid provided information about cancer/cancer treatment late effects, through educational module on effects of nicotine **Persuasion**: Use of cancer survivor testimonial to induce feelings and stimulate action in participants **Training**: Cognitive‐behavioral skills program focused on decision making, teaching theory related to quality decision making. Participants also received assistance for implementing the exercise plan **Modelling**: Use of live action videos to portray positive role modelling	**Psychological capacity**: cognitive‐behavioral skills program focused on decision making and provided balance sheet exercises in a workbook for practice **Social opportunity**: Use live action videos with diverse positive role models to illustrate decision‐making and role‐play refusal strategies **Reflective motivation**: Aimed to increase quality decision making through conscious planning and adherence to seven decision‐making criteria

Abbreviations: MVPA, moderate to vigorous physical activity; PA, physical activity.

**FIGURE 3:**
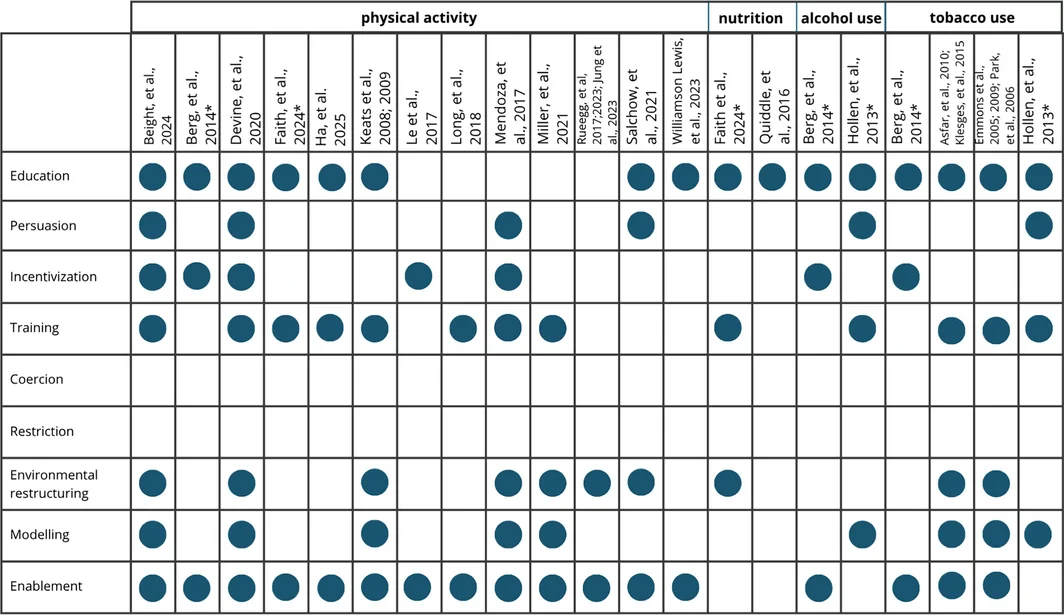
Distribution of intervention functions by behavioral domain. Interventions are represented according to their intervention domain. Interventions targeting multiple domains are therefore represented more than once, once for each relevant domain. Of the 17 distinct interventions, 13 included a physical activity component, 2 included a nutrition component, 2 included an alcohol component, and 4 included a tobacco component. Consequently, the total number of intervention representations in the figure exceeds the number of distinct interventions. Studies marked with an asterisk (*) are represented more than once because they addressed multiple intervention domains.

**FIGURE 4:**
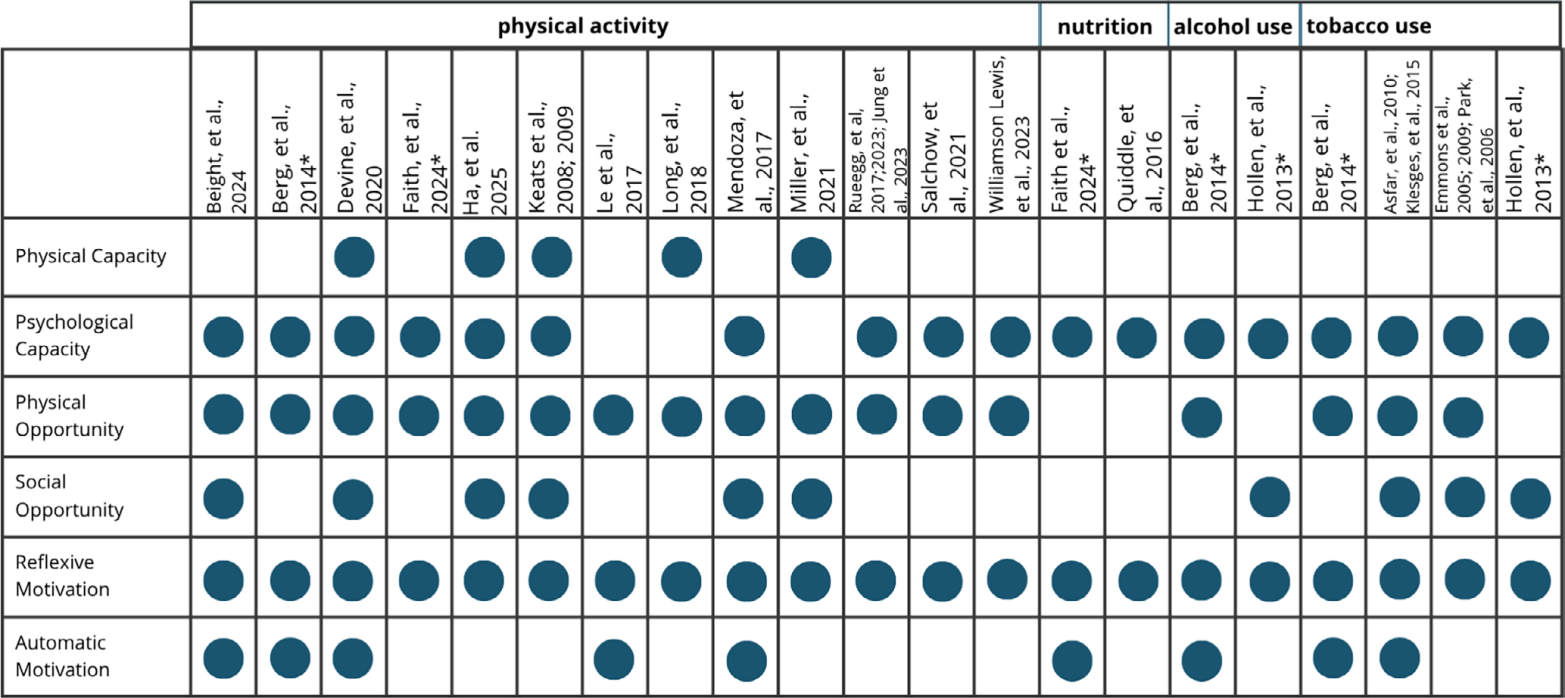
Distribution of sources of change by behavioral domain. Interventions are represented according to their intervention domain. Interventions targeting multiple domains are therefore represented more than once, once for each relevant domain. Of the 17 distinct interventions, 13 included a physical activity component, 2 included a nutrition component, 2 included an alcohol component, and 4 included a tobacco component. Consequently, the total number of intervention representations in the figure exceeds the number of distinct interventions. Studies marked with an asterisk (*) are represented more than once because they addressed multiple intervention domains.

#### 3.2.1 Physical Activity

Sixteen of the twenty‐three items [62, 66, 72, 73, 74, 78, 80, 81, 82, 83, 84, 86, 87, 88, 89, 90] extracted aimed to improve physical activity in AYA‐CCS. The 16 items reported on 13 different interventions. These interventions were developed in five different countries (USA, Switzerland, Canada, Germany, and Australia) between 2008 and 2025. These 16 items included eight randomized controlled trials [66, 72, 74, 78, 84, 87, 88, 89], two cohort studies [82, 83], two repeated longitudinal studies [80, 81], one pre‐post study [73], one cross‐sectional study [90], one randomized controlled trial research protocol [86], and one pre‐post‐trial research protocol [62].

##### 3.2.1.1 Intervention Functions

Enablement played a key role in the domain of physical activity, as all programs employed this intervention strategy. Most often, researchers provided participants with an activity tracking device [62, 66, 72, 74, 78, 82, 83, 87, 90], or access to a fitness web platform [66, 73, 74, 82, 87]. Other enablement strategies included providing a space for physical activity [80] or providing a list of local options for physical activity, such as inexpensive group classes or gyms [84, 89]. Eight interventions delivered training through imparting physical skills, with resistance or strength training [62, 74, 80, 83, 84], aerobic training [62, 74, 80, 83, 84], or flexibility training [62, 80]. Training also encompassed developing skills such as goal setting [66, 72], gradual progression [66, 72, 78], time management [78], and habit formation [62, 78]. Eight interventions provided education through educational material [72], modules [62, 73], handouts [90], or verbal education and discussion [74, 78, 80, 89], on topics including the importance [72] and benefits of physical activity [62, 80, 89, 90], risks of late effect of cancer [72], healthy and active living [62, 74, 80], physical activity recommendations or guidelines [78, 89, 90], or unspecified educational topics related to physical activity [73]. Seven interventions used environmental restructuring through changes in the physical or social context, such as altering cues to promote the behavior [74, 87], changing social norms and creating space to normalize the desired behaviors [66, 74], and providing designated areas [80] or training materials [84]. Five interventions used either incentivization or modelling. Incentivization was conveyed through gamification, such as badges, awards, and achievements [66, 72, 74], and rewards in the form of discounts on local goods and services [73], or gift cards [82]. Modelling was exhibited through peer support and modelling groups [66, 72, 74], and through enlisting survivors as mentors [80] or peer leaders [84]. Notably, only four programs used persuasion to induce positive emotions through positive reinforcement messages of peer support groups [66, 72, 74], or through individual counseling sessions recalling positive changes made by the participant [89]. None of the 13 interventions employed either coercion or restriction in the physical activity program.

##### 3.2.1.2 Sources of Behavior Change

All interventions included a component aimed at developing physical opportunity and reflective motivation. Physical opportunity was most commonly reported as being provided to access resources, whether technology, such as an activity tracker [62, 66, 72, 74, 78, 82, 83, 84, 87, 90], or through access to services [73] to promote the behaviors. It was also included as reminders to perform the behavior [72, 87, 90] or through providing training locations to perform the behavior [74, 80, 83, 84, 89]. Reflective motivation was facilitated by strategies such as goal setting [62, 66, 72, 74, 78, 80, 89, 90], habit tracking or self‐monitoring [62, 66, 72, 73, 74, 80, 82, 83, 87, 90], evaluation and reflection [74, 84, 87], and planning [78, 80, 89]. Ten interventions fostered psychological capacity through the provision of knowledge and skills [62, 66, 72, 73, 74, 78, 80, 87, 89, 90], cognitive skills and mental strategies [66, 78], decision‐making skills [78, 87], overcoming barriers [62, 80] and coping strategies [73]. Six interventions also sought to enhance social opportunities through social and peer support online communities (e.g., Facebook groups) [66, 72, 74], and enlisting peer leaders [84], survivor mentors, parents and friends [62, 80]. In addition, five programs aimed to improve automatic motivation by unconsciously driving behaviors through incentives such as badges and achievements [66, 72, 74], rewards [82], and discounted goods [73]. Lastly, five interventions improved physical capacity through physical training [62, 74, 80, 83, 84].

#### 3.2.2 Nutrition

Two [78, 85] of the 23 items extracted in this review included interventions aimed at improving the nutrition of adolescent and young adult survivors of pediatric cancer. *INAYA* [85] was conducted in 2016 and focused on improving nutrition among 23 AYA‐CCS in Germany. *INAYA* was conducted as a pilot intervention study with no control group and collected measures at baseline, at Week 2, Week 6, and Week 12. *ENHANCE* [78], an intervention conducted in 2024, aimed at improving both nutrition and physical activity in 58 survivors from the USA. *ENHANCE* was conducted as a two‐arm pilot randomized controlled trial with an education‐only control group and collected measures at baseline, after the intervention completion, and at 10–14 weeks and 22–26 weeks follow‐up.

##### 3.2.2.1 Intervention Functions

Education played a key role in both interventions and was the only intervention function used in both cases by recommendation‐based counseling, covering nutritional values, portion sizes, and relevant dietary guidelines [78, 85]. One intervention solely used education to foster behavior change [85]. For the nutrition portion of *ENHANCE*, the intervention also incorporated training (e.g., practicing satiety control and mindfulness eating), and environmental restructuring (e.g., managing social pressure to support optimal nutrition). These programs did not explicitly use persuasion, incentivization, coercion, restriction, enablement, facilitation, or modelling in attempting to change AYA's behaviors.

##### 3.2.2.2 Sources of Behavior Change

We found that both interventions called for developing psychological capacity, reflected in the imparting of skills and knowledge [78, 85]. Both programs supported reflective motivation through activities such as long‐term goal setting, monitoring progress, habit formation, time management, scheduling [78], and conscious planning [85]. One intervention fostered automatic motivation when developing skills to cope with unconscious processes influencing food choices, fostering appetite awareness, and engaging in mindfulness practices [78]. None of these two interventions targeted physical capacity, physical opportunity, or social opportunity.

#### 3.2.3 Alcohol Consumption

Two [73, 79] of the 23 records targeted alcohol consumption. Of note, both studies addressed at least one other health behavior alongside alcohol use (tobacco use [79], tobacco use and physical activity [73]). Thus, no intervention in the sample focused exclusively on alcohol consumption within this population. Berg's intervention was conducted in the USA in 2014, as a one‐arm pilot trial and collected longitudinal follow‐up data pre, post, and at a 6‐week follow‐up. *DecisionKEYS* was conducted in the USA in 2013. Researchers conducted a prospective, randomized controlled trial with a usual care control group. Data points were taken at baseline, 6 months, and 12 months post‐intervention.

##### 3.2.3.1 Intervention Functions

Both interventions called for education, providing health‐related textual and video messaging including education on the benefits of reducing alcohol consumption [73], or providing educational content with insights into the consequences of party drinking and peer pressure [79]. Moreover, one intervention utilized training by providing training in decision‐making modules aimed at providing real‐life practice of decision‐making for adolescent survivors [79]. One program used persuasion, using communication strategies to induce negative emotions by incorporating teen interviews to underscore the health consequences of substance use [79] and modelling with the use of peer modelling to promote desired behaviors [79]. One intervention used incentivization, as illustrated by the offering of deals on healthy goods and services [73], and enablement, by reducing barriers to accessing services and resources [73]. No intervention employed either coercion, environmental restructuring, or restriction.

##### 3.2.3.2 Sources of Behavior Change

Both interventions addressed psychological capability, such as imparting skills [73], decision‐making strategies [79], and reflective motivation, including strategies like habit tracking [73], or conscious planning [79]. One intervention [73] additionally incorporated elements of physical opportunity by facilitating access to relevant resources and services, as well as automatic motivation using reward‐based strategies in their intervention targeting alcohol reduction. In contrast, one intervention [79] emphasized social opportunity by leveraging peer influence. None of the studies targeting alcohol use behaviors in AYA‐CCS aimed to enhance physical capacity.

#### 3.2.4 Tobacco Consumption

Seven [70, 71, 73, 75, 76, 77, 79] of the 23 included items focused on interventions targeting tobacco use behaviors among AYA‐CCS. The seven items reported on four distinct interventions from the USA spanning from 2005 to 2014. Two of the four interventions addressed other domains of behaviors alongside tobacco use (alcohol use [91], alcohol use and physical activity [73]). One study used a one‐arm pre/post‐test design [73]; the other empirical studies reported on randomized controlled trials to test the intervention [71, 75, 76, 77, 79], or to describe the study protocol [70]. All six studies collected longitudinal data, and follow‐ups ranged from 12 weeks [73] to 12 months [71, 75, 76, 77, 79].

##### 3.2.4.1 Intervention Functions

All interventions used education as a core behavior change strategy. For instance, the programs included educational modules [73, 79], brochures [71] or educational material [75, 76, 77] to provide information such as the health risks of smoking [73], the effect of nicotine on the body [79], nicotine withdrawal and changes associated with abstinence [71], ingredients of cigarettes, as well as the cost‐benefits of quitting [76]. Three interventions used modelling through CCS peer model counseling [76], role models of childhood cancer survivors, videos of refusal strategies [79], and imparting skills through modelling [71], although they did not specify how this was achieved. Three studies employed enablement, two of which offered nicotine replacement therapy [71, 76] to reduce barriers, or deals for health services and goods were offered to participants [73] to provide access to resources. Training was employed by three interventions through practice‐based exercises imparting decision‐making skills [79] and self‐regulating and coping skills [71, 73, 76] with regard to tobacco consumption. Environmental structuring was also utilized by two interventions through altering cues to support smoking [71] and increasing social support to quit smoking [76]. One program used either persuasion, using cancer survivors to convey a positive message [79], or incentivization through offering deals and discounts at local healthy stores each time the participants completed a healthy behavior check‐in [73]. No intervention utilized coercion or restriction.

##### 3.2.4.2 Sources of Behavior Change

All interventions included a component aimed at developing psychological capability. This translated into reinforcing self‐efficacy [71, 77], addressing psychological barriers [77], preventing relapses [71, 76], supporting withdrawal tolerance [71, 77], and supporting the development of coping strategies [73]. All interventions also sought to enhance reflective motivation. For instance, programs supported decision‐making abilities [79], self‐monitoring, self‐observation [71, 73, 79] and goal setting [71, 76]. In addition, all but one intervention [79], aimed to improve physical opportunities by facilitating access to resources of health services, such as discounts on health‐related services [73], or nicotine replacement products [71, 76]. All but one program [73] also targeted social opportunities by leveraging relational and social factors, such as support from significant others through the provision of nicotine replacement products for partners [76], improving social support in preparing for cessation [71], and taking into account peer influence [79]. In contrast, only two interventions [71, 73] called for automatic motivation, notably through strategies such as removing triggers and establishing cessation rituals [71] or establishing automatic cues to provide rewards [73]. No intervention used physical capacity, such as pulmonary rehabilitation or breathing retraining in cessation contexts, as a source of the smoking cessation program.

## 4 Discussion

In this scoping review, we addressed two primary objectives. First, we identified and described interventions targeting behavior change among adolescent and young adult childhood cancer survivors, resulting in 23 items reporting on 17 distinct interventions. Together, these findings map the current landscape of behavior change interventions for AYA‐CCS, highlighting both the diversity of applied approaches and key gaps in theoretical integration, fidelity reporting, and mechanisms of action.

Our review identified 17 interventions across the four behavioral domains, but their distribution was highly uneven. The vast majority targeted physical activity (13 interventions), while far fewer focused on tobacco (four interventions), and nutrition and alcohol (2 interventions each). This imbalance is particularly concerning given that smoking, alcohol consumption, and poor nutrition represent significant cumulative risks to the long‐term health trajectory of childhood cancer survivors. This observation was also noted in other reviews with similar populations [17, 92]. Although physical activity represents an important and clinically relevant component of survivorship care, the predominance of physical activity interventions in this population highlights a potential gap in addressing other health behaviors that also contribute substantially to long‐term morbidity and mortality. It is likely that physical activity has been prioritized within survivorship research due to its consistent associations with improvements to an array of health outcomes, including cardiovascular health, insulin sensitivity, muscle mass, weight management, and lowering morbidity and mortality [26]. While inadequate physical activity is estimated to account for approximately 8% of all‐cause mortality [93] and substantially increases the risk of comorbid conditions [94], the global burden attributable to other behaviors is equally striking. Poor diet is a leading contributor to all‐cause mortality and disability [35], alcohol use is responsible for around 5% of all‐cause mortality and 5% of all‐cause morbidity worldwide [38], and tobacco remains one of the single most preventable causes of death [95], contributing to roughly 20% of all‐cause mortality in men and 6% in women [44]. Given these figures, the disproportionate focus on physical activity is problematic. Moreover, these behaviors often co‐occur, such that risk in one domain clusters with risk in others, compounding health consequences [4]. A few hypotheses could be formulated to account for this imbalance. It may refer to differences in measurement feasibility, the longer‐standing history of physical activity research in oncology populations, perceived clinical relevance, and potential ethical or regulatory considerations. However, these factors remain hypotheses that require further investigation. This is a critical gap in the evidence base, suggesting that interventions in AYA‐CCS populations should be broadened to more systematically address multiple lifestyle behaviors.

The presence of multi‐domain interventions, though uncommon, constituted a second notable feature. Three examples were identified: targeting physical activity, alcohol, and tobacco [73]; tobacco and alcohol reduction [79]; and physical activity and nutrition [78]. The effectiveness of multi‐domain behavior change interventions compared with single‐domain interventions is inconclusive, with mixed findings reported in the broader literature [96]. Prior research indicates that these approaches may systematically differ not only in the number of behaviors addressed, but also in the type and quantities of behavior change techniques employed [96]. In the present review, it was unclear whether the behavior change techniques employed were systematically matched to each targeted domain. For example, the intervention function *training* cannot be applied in the same way to physical activity and alcohol use. Training could be implemented for increasing physical activity by developing physical skills, whereas reducing alcohol consumption may require training focused on cognitive skills and coping strategies. Therefore, greater planning of multi‐domain interventions is needed to ensure that appropriate behavior change techniques are aligned with the target behavior, and that intervention designs are tailored to the specific behavioral domain [97].

Another important finding concerns the type of intervention studies reported. More than half (60%) were feasibility studies, with few addressing proof‐of‐concept (Phase IIa, ORBIT model) or progressing toward efficacy trials (Phase III, ORBIT model). This pattern indicates a relative immaturity of the evidence base. The ORBIT model framework outlines a phased process from development through refinement to eventual efficacy evaluation [69]. Within this framework, most identified studies were positioned in earlier phases, indicating that many interventions remain in development or optimization stages. Overall, these findings describe the current maturity of the evidence base for behavioral interventions in AYA‐CCS, highlighting that relatively few interventions have progressed to later‐stage efficacy testing.

We identified clear challenges to intervention evaluation and reproducibility. Descriptions of intervention materials were often incomplete, a reporting gap commonly observed in the literature [98], with many studies listing only broad categories of resources while omitting critical details about their content, format, or delivery. This is a significant limitation, as intervention materials (e.g., counseling scripts, training manuals, or digital platforms) represent the tangible components that practitioners and participants interact with. Without access to such information, interventions may not be implemented consistently, fidelity cannot be assessed, and future adaptations risk drifting away from the original design. The fact that only one study has made a training manual publicly available highlights how rarely materials are made fully accessible. Improving transparency in this area is critical and would directly support replication, scalability, and uptake.

Reporting on the contextual aspect of delivery (such as setting) was often incomplete, although such information is considered essential, as setting and environmental factors can significantly influence the feasibility and effectiveness of the interventions [67]. It is well‐known that intervention providers affect intervention outcomes, especially in programs that rely heavily on communication [67]. Clarity about provider characteristics is crucial to ensure that interventions are delivered by appropriately trained personnel and can be replicated across comparable contexts. Recent methodological guidance emphasizes that assessing and transparently reporting on facilitator competence and fidelity is essential to understanding how and why interventions achieve their effects [99]. Accordingly, interventions should clearly describe who delivered the program, including their training and qualifications, how provider competence was assessed before and during delivery, and whether delivery was part of the provider's professional role or specific to the study. Such reporting not only strengthens confidence in study findings but also supports feasibility, scalability, and fidelity assessments across settings, thereby advancing the translation of research into effective, real‐world practice [100].

Our review underscores a persistent gap in fidelity monitoring within health behavioral interventions. In the present review, most studies relied solely on participant attendance as a proxy for fidelity, which reflects exposure but not whether the intervention was delivered as intended. It is important that researchers realize that without data on implementation quality, protocol adherence, or delivery consistency, it is speculative to attribute outcomes to the intervention itself. Comprehensive fidelity reporting should include both planned strategies, such as provider training, manuals, and supervision, and the actual implementation of these strategies [67].

### 4.1 Behavior Change Techniques

Based on the BCW, we identified intervention functions and sources of behavior change in all 17 interventions. Most interventions provided a clear rationale, often grounded in well‐established behavior change theories, a notable strength, as grounding interventions in theory helps to identify the mechanisms through which change occurs and underpins the components of an intervention that are essential rather than optional or incidental [67]. However, one quarter of the studies did not draw on any explicit theory. This lack of theoretical grounding raises significant challenges for the field, as it limits the ability to understand how and why behavior change is expected to occur, to select appropriate intervention strategies and behavior change techniques, and to interpret why an intervention succeeds or fails [97]. Consequently, it makes findings more difficult to interpret, reduces the potential for replication, and may contribute to limited intervention effectiveness. Most interventions described tailoring strategies to individual needs, such as motivational stage, behavioral profile, or physical limitations. Thoroughly detailing the tailoring of an intervention is instrumental, as not all participants receive an identical intervention when tailored [101]; thus, providing details on how and why the intervention was modified to suit participants is crucial [67]. However, some reports omitted key details, such as the required time commitment or the parameters for tailoring. Without this information, it is difficult to figure out challenges for future adaptations, and practitioners cannot confidently reproduce the intervention. Consequently, researchers may have difficulty assessing whether individual differences in outcomes in studies are due to the intervention itself or to variation in its delivery.

Within this pattern, interventions frequently relied on providing information through counseling, written resources, or didactic sessions, assuming that increased knowledge will naturally translate into improved health practices [102, 103]. However, evidence from health literacy research suggests that understanding recommendations is not equivalent to being able to apply them effectively in real‐life contexts [102, 104, 105]. While these interventions can effectively enhance psychological capability through knowledge transfer and skill development, their reliance on informational approaches alone provides only limited engagement with the motivational and contextual mechanisms necessary to support sustained behavior maintenance over time [102].

A consistent feature across domains was the predominance of supportive, non‐coercive strategies over directive or restrictive approaches. This preference for supportive approaches likely reflects the developmental needs of AYAs, who value autonomy, self‐direction, and the avoidance of external control [106].

A key observation is that most interventions rely heavily on extrinsic motivation, with limited use of intrinsic strategies. This is critical, as behaviors aligned with autonomy, competence, and relatedness are more likely to be sustained over time [107]. Ineffective outcomes may stem from poorly targeted motivational approaches [108]. Additionally, physical capability and automatic motivation were underutilized, highlighting missed opportunities to enhance intervention impact through more comprehensive motivational targeting. In broader populations, interventions incorporating skill‐building or structured physical activity may be more effective than education‐only formats in improving self‐efficacy and adherence in young people [109, 110, 111]. Its limited integration is concerning, given its role in sustaining behavioral patterns [107]. These gaps highlight missed opportunities to enhance intervention impact through more comprehensive motivational targeting.

Another salient feature across domains was the integration of multiple intervention functions and sources of behavioral change. By activating multiple components, interventions may achieve synergistic reinforcement, thus considerably increasing the likelihood of producing durable change. However, evidence also suggests that the number of behavior change techniques used does not predict the effectiveness of an intervention [112, 113, 114]. Similarly, a review in AYA survivors of cancer reported that the number of behavior change techniques within an intervention does not necessarily enhance its impact [19]. It is also difficult to compare large numbers of behavior change techniques or their combinations, and interventions often include techniques that add little to meaningful behavior change [115]. These findings emphasize that the effectiveness of an intervention depends less on the sheer number of behavior change techniques and more on selecting a small, theoretically grounded set of techniques that directly target the mechanisms of change. Clearly specifying these techniques early in the intervention development process strengthens transparency, improves replicability, and reinforces the causal pathway between theory and behavioral outcomes, ultimately supporting the design of more effective interventions [19].

### 4.2 Physical Activity

Many studies solely provided activity trackers with basic instructions, reiterating known exercise benefits rather than addressing the complex challenge of sustaining long‐term behavior change. In a recent systematic review, Coffman et al. [19] similarly reported that, although widely used, activity trackers were not effective as standalone tools for behavior change. Previous intervention studies in the general population suggest that standalone activity tracker interventions may be insufficient to produce meaningful behavior change, with greater effects observed when they are combined with other behavior change strategies [116, 117, 118]. Furthermore, few interventions included supervised training to build physical capacity, relying instead on unsupervised, home‐based activity, which often fails to support sustained engagement [119]. This is at odds with findings in broader oncology populations, where supervised, multimodal programs consistently yield greater improvements in strength, functioning, and quality of life [120, 121]. Current interventions may therefore undermine sustainability by neglecting physical capacity and underutilizing key techniques related to automatic motivation [19], social opportunity [19], and structured training [119].

### 4.3 Nutrition

Previous evidence from health interventions in adolescent and young adult populations shows that strategies beyond education, such as persuasive messaging [122], financial incentives [123], peer modelling [124], and habit‐forming enablement [125, 126], are effective in improving dietary behaviors, whereas education alone produces modest, short‐lived effects [127, 128]. Thus, these previous findings suggest that several intervention functions beyond education are not only commonly used with healthy AYAs but are also effective in shaping dietary behaviors [129, 130, 131, 132]. However, these evidence‐based practices have not been translated into interventions for the AYA‐CCS population, leaving important contextual and motivational determinants of sustained dietary behavior change unaddressed.

### 4.4 Alcohol Consumption

The very limited evidence base is striking given that alcohol use remains a major health risk among AYA cancer survivors [42, 133, 134]. The interventions primarily employed education and training, emphasizing knowledge, risk awareness, and coping skills, with the occasional use of emotional appeals, role models, or incentives. The focus on individual knowledge and skills contrasts with previous evidence showing that social‐norms interventions [135], peer‐led strategies [136, 137], and multilevel approaches [138] more effectively reduce alcohol consumption among youth. Since alcohol use in adolescence is strongly shaped by social norms [139, 140], survivorship programs that neglect social and contextual influences risk limited impact. This gap highlights the importance of integrating socially embedded and context‐sensitive strategies into survivorship care to meaningfully address alcohol use in this population.

### 4.5 Tobacco Consumption

Few interventions targeted automatic motivation, despite nicotine addiction being strongly shaped by conditioned cues and cravings [141, 142]. Without strategies that address cravings, habit loops, and environmental cues, relapse risk remains high. Moreover, the emphasis of most interventions remained on equipping individuals with knowledge and access to cessation aids, approaches that, while supportive, may be insufficient to counter the deeply habitual and socially reinforced nature of nicotine dependence [143, 144].

### 4.6 Clinical Implications

Overall, this review not only catalogues existing interventions but also identifies critical evidence gaps that clarify where research is concentrated, where it is lacking, and how it can be strengthened to inform more balanced, theory‐driven, and clinically relevant survivorship interventions for AYA‐CCS. From a clinical perspective, these findings indicate several priorities. First, survivorship care for AYA‐CCS should extend beyond physical activity to systematically address nutrition, alcohol use, tobacco use, and other lifestyle behaviors for which evidence in this population is scarce but robust in healthy adolescent and young adult populations. Second, clinicians should prioritize evidence‐based behavior change techniques, rather than relying predominantly on education alone, when counseling AYA‐CCS about health behaviors. Third, the review underscores the importance of manualized, well‐described programs with clearly specified behavior change techniques and underlying mechanisms of action. Developing and disseminating intervention manuals, training materials, and standard operating procedures would facilitate implementation in new centers, support adaptations to local settings and enhance fidelity across sites.

### 4.7 Limitations

Several limitations inherent to our review process should be acknowledged. First, because few interventions explicitly specified their behavior change techniques, the mapping of strategies necessarily relied on inference, limiting the precision, consistency, and systematicity of our analysis and possibly introducing interpretive bias. Mapping intervention content to Michie et al.'s [64] BCW model was based on interpretation of reported intervention descriptions, as most primary studies did not explicitly state the behavior change techniques or theoretical constructs used. While established taxonomies and coding guidance were applied to enhance consistency, this process necessarily involved a degree of subjective judgment in inferring intervention functions and sources of behavior change from often incomplete or variably reported intervention details. As a result, the mappings presented should be understood as interpretative representations rather than definitive classifications of the original intervention design. Second, limited descriptions of intervention content constrained our ability to capture the full complexity of behavioral strategies. As interventions in practice are often more complex than final manuscripts suggest, this constrained our ability to capture the full range of behavioral strategies and to identify underlying mechanisms of change and their relative impacts. Third, although formal risk‐of‐bias assessment was not undertaken in this scoping review, in line with JBI guidance [54], we acknowledge that variability in methodological quality across the included studies may influence the interpretation of the evidence. The included literature is predominantly composed of feasibility and early‐phase studies, many of which are characterized by small sample sizes, heterogeneous study designs, limited use of control groups, and incomplete reporting of intervention components and fidelity. In addition, variability in outcome measurement and follow‐up duration further limits comparability across studies. These methodological constraints should be considered when interpreting the findings, as they preclude conclusions regarding comparative effectiveness or efficacy of interventions. Rather, the results should be understood as a descriptive synthesis of the available evidence, highlighting patterns in intervention design and reporting as well as important gaps for future, more rigorously designed, adequately powered, and methodologically transparent intervention studies in adolescent and young adult childhood cancer survivorship research.

A key limitation of the included evidence is the inconsistent and often incomplete reporting of important participant characteristics across studies. Variables such as age at diagnosis, age at intervention, cancer type, and treatment history were not uniformly reported, which restricts the ability to fully characterize the study populations. As a result, it is difficult to determine the extent to which the included samples are comparable to one another or representative of the broader population of interest. This limitation has important implications for generalizability. Without detailed and consistent participant information, it is unclear whether the findings apply equally across different survivor subgroups, particularly given the known heterogeneity in cancer types, treatment exposures, and developmental stages at diagnosis. Consequently, the external validity of the synthesized evidence is reduced, and caution is required when extending conclusions beyond the specific populations represented in the included studies. In addition, missing participant information limits the interpretation of intervention effectiveness. The absence of key clinical and demographic descriptors makes it difficult to assess whether variability in reported outcomes reflects true differences in intervention impact or underlying differences in participant characteristics across studies. This also restricts the ability to identify potential effect modifiers or to interpret subgroup differences, thereby limiting insight into for whom and under what conditions interventions may be most effective.

The included studies were geographically concentrated, with most conducted in high‐income countries, 61% of them conducted in the United States. The current portrait may not be applicable to other countries or settings, as differences in healthcare systems, survivorship care infrastructure, and access to rehabilitation or preventive services may influence both the design and delivery of interventions, as well as participant engagement and outcomes [145, 146, 147]. In addition, cultural norms and societal attitudes towards lifestyle behaviors (e.g., physical activity, diet, and health promotion) vary across settings and may shape both baseline behaviors and responsiveness to intervention strategies [145, 148].

Finally, our synthesis was constrained as many of the included tobacco‐cessation interventions were developed prior to the widespread adoption of e‐cigarettes and vaping among AYA, which limits the contemporary relevance of our findings. Because vaping involves distinct behavioral patterns, cues, and social dynamics, strategies effective in the mid‐2000s may have reduced relevance today, a concern amplified by the sharp rise in adolescent vaping in recent years (with prevalence reaching 21.3% among 15–17‐year‐olds) [149]. While vaping is still an emerging area of research [150], recent studies indicate that nearly half of young adult cancer survivors report having used e‐cigarettes, and about a quarter are current users [151]. Although research is still limited, existing literature suggests that vaping behaviors among survivors follow trends observed in the general population and pose a potential long‐term health threat [150].

Collectively, these methodological constraints highlight the need for improved reporting standards, more detailed intervention descriptions, and updated evidence that reflects current tobacco‐use behaviors and contexts.

## 5 Conclusion

In this scoping review of 23 items reporting on 17 distinct interventions, we mapped the behavioral domains, theoretical foundations, and behavior change techniques employed in interventions targeting adolescent and young adult childhood cancer survivors. This scoping review offers the first comprehensive synthesis of behavior change interventions targeting AYA‐CCS across multiple lifestyle domains. Our findings reveal a marked imbalance, with nutrition‐ and alcohol‐related interventions particularly underrepresented relative to physical activity research. By mapping interventions using the BCW, the review provides a systematic account of the functions and sources of behavior change employed, highlighting not only conceptual gaps (e.g., underuse of physical capability and automatic motivation) but also clear opportunities to enhance intervention impact. The findings reveal key gaps that constrain theoretical integration, intervention comparability, and the advancement of cumulative evidence in this field. First, although most interventions reported a theory‐based rationale, few explicitly articulated how theoretical constructs guided the selection or application of specific behavior change techniques. Theoretical integration often remained superficial, limiting the capacity to interpret outcomes, identify mechanisms of change, and assess coherence between intervention design and expected processes. Future research should strengthen the explicit alignment between theoretical models and the operationalization of intervention components, ensuring that the rationale for technique selection is clearly justified and reported. Second, the evidence base remains disproportionately concentrated on physical activity, with relatively few interventions addressing other domains of health. Given that these behaviors frequently co‐occur and exert cumulative effects on long‐term health, there is a need for multi‐behavioral approaches that more comprehensively reflect the risk profiles of AYA‐CCS. Third, the reporting of intervention content, materials, and fidelity was frequently incomplete, limiting transparency, replication, and the identification of active components driving behavioral outcomes. Enhancing the systematic reporting, using tools such as the TIDieR checklist [67, 68] list, will be essential to strengthen methodological rigour, reproducibility, and transparency around treatment fidelity, including key contextual and provider‐related factors that influence intervention delivery and effectiveness. Finally, most interventions remain at an early developmental stage, with few advancing to large‐scale efficacy or effectiveness testing. Progressing beyond feasibility work toward well‐powered trials that assess mechanisms of action, implementation fidelity, and long‐term maintenance is critical to establish a robust evidence base. Taken together, these findings highlight the need for interventions that are more theory‐driven, clearly reported, and methodologically robust. These should address multiple health behaviors and include mechanisms that promote adherence and long‐term maintenance for this vulnerable population.

## Author Contributions

**C. Gélinas‐Gagné:** conceptualization, data curation, formal analysis, writing – review and editing, writing – original draft, investigation, validation, visualization. **K. Bilodeau:** conceptualization, formal analysis, supervision, writing – original draft, writing – review and editing, investigation, validation. **N. Guarascio:** conceptualization, data curation, writing – review and editing, formal analysis, investigation. **M. Caru:** conceptualization, formal analysis, writing – review and editing. **H. Coltin:** conceptualization, formal analysis, writing – review and editing. **D. Curnier:** conceptualization, formal analysis, writing – review and editing. **L. Desjardins:** conceptualization, formal analysis, writing – review and editing. **C. Laverdière:** conceptualization, formal analysis, writing – review and editing. **V. Marcil:** conceptualization, formal analysis, writing – review and editing. **R. Santiago:** conceptualization, formal analysis, writing – review and editing. **S. Vairy:** conceptualization, formal analysis, writing – review and editing. **S. Sultan:** conceptualization, data curation, investigation, validation, formal analysis, supervision, writing – original draft, writing – review and editing.

## Funding

Fonds de Recherche du Québec (FRQ) doctoral research scholarship (B2) (to Charlotte Gélinas‐Gagné, 2026–2030), Cole Foundation (Cole Doctoral Fellowship 2025–2027 to Charlotte Gélinas‐Gagné), Laboratoire Bilodeau, Research Center Hôpital Maisonneuve‐Rosemont, Fondation CHU Sainte‐Justine, Fondation Charles‐Bruneau.

## Conflicts of Interest

The authors declare no conflicts of interest.

## Supporting information


**Data S1:** cnr270721‐sup‐0001‐Supinfo.docx.
**Supplementary File 1**: Summary of the modifications made to the original review protocol, including changes to the target population, inclusion of general adult interventions, and grey literature sources, with corresponding rationales.
**Supplementary File 2**: Full electronic search strategy.
**Supplementary File 3**: Preferred Reporting Items for Systematic reviews and Meta‐Analyses extension for Scoping Reviews (PRISMA‐ScR) checklist, including the corresponding page numbers where each reporting item is addressed in the manuscript.

## Data Availability

The data that support the findings of this study are available from the corresponding author upon reasonable request.
