# Supplementary material for: Behavior Change Interventions to Promote Healthy Lifestyles Among Adolescent and Young Adult Childhood Cancer Survivors: A Scoping Review
Source: Cancer Rep (Hoboken). 2026 Oct 18;9(10):e70721. doi: 10.1002/cnr2.70721 (PMC13677448; doi:10.1002/cnr2.70721)
Supplement: Supplementary file 1 [file CNR2-9-e70721-s001.docx]

## Supplementary File 1

## Protocol Modifications

| **Protocol Component** | **Original Protocol** | **Modification** | **Rationale** |
| --- | --- | --- | --- |
| **Target population (age range)** | Adolescents and young adults (AYA), 15–39 years | Lower boundary revised to 13 years; upper boundary of 39 years retained but refined with the research team | Aligns with definitions of adolescence commonly used in oncology and QoL research; ensures inclusion of early adolescents while maintaining consistency with prevailing AYA definitions |
| **Inclusion of general adult interventions** | Not specified explicitly | Excluded interventions designed for general adult populations without AYA-specific adaptation | Such programs do not address AYA-specific needs; inclusion would compromise relevance and transferability of findings |
| **Grey literature sources** | Planned inclusion of abstracts, case reports, short communications, theses, letters to the editor, and protocol papers | Grey literature excluded | Behaviour change techniques and detailed intervention descriptions are rarely reported in grey literature; exclusion ensures systematic extraction aligned with review objectives |

***Supplementary File 1:*** *Summary of the modifications made to the original review protocol, including changes to the target population, inclusion of general adult interventions, and grey literature sources, with corresponding rationales.*

## Supplementary File 2

## Search Strategy

| **Population*** | **AND** | **Oncology*** | **AND** | **Condition*** | **AND** | **Intervention*** |
| --- | --- | --- | --- | --- | --- | --- |
| child* OR  youth OR  adolescen*  OR teen*  OR  tween* OR  AYA OR  "young adult*" OR  pediatric* OR  paediatric* |  | cancer* OR  neoplasm* OR  carcinom* OR  lymphom* OR  leukemia* OR  leukaemia* OR  tumor* OR  tumour* OR  oncolog* OR  chemo OR  chemotherapy OR  "cancer treatment*" OR  "radiation therapy" OR  radiotherapy OR  "radiological therapy" |  | survivor* OR  remission OR  recovery OR  recovered OR  aftercare OR  "after care" OR  "after treatment" OR  "post treatment" OR  "post therapy" OR  "post chemo*" OR  "post cancer" OR  "long-term care" |  | program* OR  intervention* OR  promot* OR  modification OR  change OR  habit* OR  "behavio* change" OR lifestyle OR  "life style" |

**AND**

| **Domain of Physical Activity** | **OR** | **Domain of Nutrition** | **OR** | **Domain of Alcohol use** | **OR** | **Domain of Tobacco use** |
| --- | --- | --- | --- | --- | --- | --- |
| "physical activit*" OR "activity level*" OR "physical training" OR "aerobic training" OR aerobics OR  "athletic training" OR  exercis* OR  workout* OR  fitness OR  sport* OR  sedentar* |  | nutrition* OR  malnutrition OR  nutrient* OR  diet* OR  meal* OR  food* OR  eating OR  "eating behavio*" |  | alcohol OR  "alcohol* usage" OR "alcohol use" OR  "alcohol intake" OR  "alcohol consumption" OR  "alcohol abuse" OR  drinking OR  "excessive drinking" OR "binge drinking" OR  "drinking behavio*" |  | tobacco OR  cigarette* OR  smoking |

### [(exp Child/ or Adolescent/ or Young Adult/ or Pediatrics/ or (child* or youth or adolescen* or teen* or tween* or AYA or "young adult*" or pediatric* or paediatric*).ti,ab,kw.) AND (exp Neoplasms/ or exp Leukemia/ or exp Lymphoma/ or exp Brain Neoplasms/ or (cancer* or neoplasm* or carcinom* or lymphom* or leukemia* or leukaemia* or tumor* or tumour* or oncolog* or chemo or chemotherapy or "cancer treatment*" or "radiation therapy" or radiotherapy or "radiological therapy").ti,ab,kw.) AND (exp Cancer Survivors/ or exp Continuity of Patient Care/ or Long-Term Care/ or (survivor* or remission or recovery or recovered or aftercare or "after care" or "after treatment" or "post treatment" or "post therapy" or "post chemo*" or "post cancer" or "long-term care").ti,ab,kw.) AND (exp Health Education/ or exp Health Behavior/ or exp Life Style/ or (program* or intervention* or promot* or modification or change or habit* or "behavio* change" or lifestyle or "life style").ti,ab,kw.)] AND [(exp Motor Activity/ or exp Physical Fitness/ or exp Sports/ or ("physical activit*" or "activity level*" or "physical training" or "aerobic training" or aerobics or "athletic training" or exercis* or workout* or fitness or sport* or sedentar*).ti,ab,kw.) OR (exp Diet/ or exp Food/ or (nutrition* or malnutrition or nutrient* or diet* or meal* or food* or eating or "eating behavio*").ti,ab,kw.) OR (exp Drinking Behavior/ or (alcohol or drinking or "alcohol* usage" or "alcohol use" or "alcohol intake" or "alcohol consumption" or "alcohol abuse" or "excessive drinking" or "binge drinking" or "drinking behavio*").ti,ab,kw.) OR (exp Smoking/ or exp "Tobacco Use"/ or exp Tobacco Products/ or Smoking Cessation/ or Smoking Reduction/ or (tobacco or cigarette* or smoking).ti,ab,kw.)]

***Supplementary File 2:*** Full electronic search strategy.

## Supplementary File 3

**Preferred Reporting Items for Systematic reviews and Meta-Analyses extension for Scoping Reviews (PRISMA-ScR) Checklist**

| **SECTION** | **ITEM** | **PRISMA-ScR CHECKLIST ITEM** | **REPORTED ON PAGE #** |
| --- | --- | --- | --- |
| **TITLE** | | | |
| Title | 1 | Identify the report as a scoping review. | Multiple mentions (including in the title and 1^st^ paragraph of the method (p.8) |
| **ABSTRACT** | | | |
| Structured summary | 2 | Provide a structured summary that includes (as applicable): background, objectives, eligibility criteria, sources of evidence, charting methods, results, and conclusions that relate to the review questions and objectives. | p.4 |
| **INTRODUCTION** | | | |
| Rationale | 3 | Describe the rationale for the review in the context of what is already known. Explain why the review questions/objectives lend themselves to a scoping review approach. | p.7-8 |
| Objectives | 4 | Provide an explicit statement of the questions and objectives being addressed with reference to their key elements (e.g., population or participants, concepts, and context) or other relevant key elements used to conceptualize the review questions and/or objectives. | p.7 |
| **METHODS** | | | |
| Protocol and registration | 5 | Indicate whether a review protocol exists; state if and where it can be accessed (e.g., a Web address); and if available, provide registration information, including the registration number. | p.8 |
| Eligibility criteria | 6 | Specify characteristics of the sources of evidence used as eligibility criteria (e.g., years considered, language, and publication status), and provide a rationale. | p.8-10 |
| Information sources* | 7 | Describe all information sources in the search (e.g., databases with dates of coverage and contact with authors to identify additional sources), as well as the date the most recent search was executed. | p.11 |
| Search | 8 | Present the full electronic search strategy for at least 1 database, including any limits used, such that it could be repeated. | (supp. File) |
| Selection of sources of evidence† | 9 | State the process for selecting sources of evidence (i.e., screening and eligibility) included in the scoping review. | p.10-11 |
| Data charting process‡ | 10 | Describe the methods of charting data from the included sources of evidence (e.g., calibrated forms or forms that have been tested by the team before their use, and whether data charting was done independently or in duplicate) and any processes for obtaining and confirming data from investigators. | p. 11-12 |
| Data items | 11 | List and define all variables for which data were sought and any assumptions and simplifications made. | Figure 1 |
| Critical appraisal of individual sources of evidence§ | 12 | If done, provide a rationale for conducting a critical appraisal of included sources of evidence; describe the methods used and how this information was used in any data synthesis (if appropriate). | NA |
| Synthesis of results | 13 | Describe the methods of handling and summarizing the data that were charted. | p.12-13 |
| **RESULTS** | | | |
| Selection of sources of evidence | 14 | Give numbers of sources of evidence screened, assessed for eligibility, and included in the review, with reasons for exclusions at each stage, ideally using a flow diagram. | p.14 |
| Characteristics of sources of evidence | 15 | For each source of evidence, present characteristics for which data were charted and provide the citations. | Table 1 |
| Critical appraisal within sources of evidence | 16 | If done, present data on critical appraisal of included sources of evidence (see item 12). | NA |
| Results of individual sources of evidence | 17 | For each included source of evidence, present the relevant data that were charted that relate to the review questions and objectives. | Table 1 |
| Synthesis of results | 18 | Summarize and/or present the charting results as they relate to the review questions and objectives. | Table 2-3 |
| **DISCUSSION** | | | |
| Summary of evidence | 19 | Summarize the main results (including an overview of concepts, themes, and types of evidence available), link to the review questions and objectives, and consider the relevance to key groups. | p. 15-22 |
| Limitations | 20 | Discuss the limitations of the scoping review process. | p.33-35 |
| Conclusions | 21 | Provide a general interpretation of the results with respect to the review questions and objectives, as well as potential implications and/or next steps. | p.34-35 |
| **FUNDING** | | | |
| Funding | 22 | Describe sources of funding for the included sources of evidence, as well as sources of funding for the scoping review. Describe the role of the funders of the scoping review. | p.3 |

JBI = Joanna Briggs Institute; PRISMA-ScR = Preferred Reporting Items for Systematic reviews and Meta-Analyses extension for Scoping Reviews.

* Where *sources of evidence* (see second footnote) are compiled from, such as bibliographic databases, social media platforms, and Web sites.

† A more inclusive/heterogeneous term used to account for the different types of evidence or data sources (e.g., quantitative and/or qualitative research, expert opinion, and policy documents) that may be eligible in a scoping review as opposed to only studies. This is not to be confused with *information sources* (see first footnote).

‡ The frameworks by Arksey and O’Malley (6) and Levac and colleagues (7) and the JBI guidance (4, 5) refer to the process of data extraction in a scoping review as data charting*.*

§ The process of systematically examining research evidence to assess its validity, results, and relevance before using it to inform a decision. This term is used for items 12 and 19 instead of "risk of bias" (which is more applicable to systematic reviews of interventions) to include and acknowledge the various sources of evidence that may be used in a scoping review (e.g., quantitative and/or qualitative research, expert opinion, and policy document).

*From:* Tricco AC, Lillie E, Zarin W, O'Brien KK, Colquhoun H, Levac D, et al. PRISMA Extension for Scoping Reviews (PRISMAScR): Checklist and Explanation. Ann Intern Med. 2018;169:467–473. [doi: 10.7326/M18-0850](http://annals.org/aim/fullarticle/2700389/prisma-extension-scoping-reviews-prisma-scr-checklist-explanation).

***Supplementary File 3:*** *Preferred Reporting Items for Systematic reviews and Meta-Analyses extension for Scoping Reviews* *(PRISMA-ScR) checklist, including the corresponding page numbers where each reporting item is addressed in the manuscript.*
